# Supplementary material for: Combined metabolomics and proteomics analysis of vascular cognitive impairment in hypertensive rats induced by endothelial injury
Source: PLoS One. 2025 Oct 9;20(10):e0332827. doi: 10.1371/journal.pone.0332827 (PMC12510566; doi:10.1371/journal.pone.0332827)
Supplement: Attachment — Submitted filename: PONE-D-24-59603_reviewer.pdf [file pone.0332827.s006.pdf]

PLOS ONE

Combined metabolomics and proteomics analysis of vascular cognitive impairment in hypertensive rats induced by endothelial injury

--Manuscript Draft--

**Manuscript Number:**	PONE-D-24-59603
**Article Type:**	Research Article
**Full Title:**	Combined metabolomics and proteomics analysis of vascular cognitive impairment in hypertensive rats induced by endothelial injury
**Short Title:**	Metabolomics-proteomics analysis of vascular cognitive impairment rats
**Corresponding Author:**	Jiangang Liu<br>China Academy of Chinese Medical Sciences Xiyuan Hospital<br>Bei Jing, CHINA
**Keywords:**	vascular cognitive impairment; vascular dementia; hypertensive rats; hypertension; endothelial dysfunction; metabolomics; proteomics
**Abstract:**	Aim To study the molecular biological mechanism of vascular cognitive impairment model rats induced by hypertension combined with endothelial injury based on metabolomics and proteomics, and to find proteins or metabolites related to key metabolic pathways.<br>Methods SPF hypertensive rats (SHR) were used to damage the common carotid artery endothelium with microcurrent to induce vascular cognitive impairment model in rats (Model group), SHR rats (SHR group) and SPF normotensive Wistar-Kyoto rats (WKY) with the same genetic background were used as sham operation groups (no current stimulation after dissection), with 10 rats in each group. Morris water maze, PNT experiment and SPT experiment were used to detect the learning and memory ability of rats. TMT quantitative proteomics technology combined with liquid chromatography tandem mass spectrometry (LC-MS/MS) was used to detect the difference in metabolites and proteins in brain tissue of rats with vascular cognitive impairment.<br>Results From Day 1 to 5, compared with the WKY group, both the Model and SHR groups exhibited shortened incubation periods and slower average swimming speeds, with statistical differences ($P<0.01$); On Day 6, the number of Model group crossings was significantly reduced ($P<0.05$), the time spent searching for the target quadrant was reduced ($P<0.05$), and the total distance traveled by the target quadrant was reduced ($P<0.05$). The pathological results showed that compared with the WKY group, the number of glial cells and neuronal cells in the brain hippocampus of rats in the SHR group and the Model group was reduced, the cell bodies were relatively loosely arranged, and Nissl body staining was lighter. The morphology of some hippocampal neurons in the Model group changes from granular to powdery, The morphology of some hippocampal neurons in the Model group changed from granular to powdery, with nuclear consolidation, accompanied by cellular edema and necrosis. The metabolomics results showed that there were 437 significantly different metabolites between the Model group and the WKY group, 128 (+) and 309 (-); there were 449 significantly different metabolites between the Model group and the SHR group, 119 (+), 330(-). The differential metabolites in each group were mainly concentrated in metabolic pathways such as alanine, aspartate and glutamate metabolism, arginine and proline metabolism, and amino acid biosynthesis. The proteomics results showed that compared with the WKY group, there were 141 differentially expressed proteins in the Model group, 55 (+), and 86 (-). Pathways with higher connectivity in the action network include "glycine, serine and threonine metabolism", "phenylalanine metabolism", etc.; compared with the SHR group, there were 28 differentially expressed proteins in the Model group and 15 (+). 13 species (-). The significantly enriched pathways are "focal adhesion" and "relaxin signaling pathway".<br>Conclusion Rats with hypertension combined with endothelial injury have behavioral disorders, which are similar to the causes and signs of clinical vascular cognitive impairment; the biological mechanisms manifested in the model involve amino acid metabolism disorders, relaxin signaling pathway and impaired focal adhesion function.

Powered by Editorial Manager® and ProduXion Manager® from Aries Systems Corporation

Order of Authors:	Xiao Zhang<br>Surui Chang<br>Jiangang Liu<br>Xiaoyuan Wang
Additional Information:	
Question	Response
**Financial Disclosure**<br><br>Enter a financial disclosure statement that describes the sources of funding for the work included in this submission. Review the submission guidelines for detailed requirements. View published research articles from *PLOS ONE* for specific examples.<br><br>This statement is required for submission and **will appear in the published article** if the submission is accepted. Please make sure it is accurate.<br><br>**Funded studies**<br>Enter a statement with the following details:<br>• Initials of the authors who received each award<br>• Grant numbers awarded to each author<br>• The full name of each funder<br>• URL of each funder website<br>• Did the sponsors or funders play any role in the study design, data collection and analysis, decision to publish, or preparation of the manuscript?<br><br>Did you receive funding for this work?	Yes
Please add funding details.<br>as follow-up to "**Financial Disclosure**<br><br>Enter a financial disclosure statement that describes the sources of funding for the work included in this submission. Review the submission guidelines for detailed requirements. View published research articles from *PLOS ONE* for specific examples.<br><br>This statement is required for submission	This study was supported by Major Increase and Decrease Projects at Central Level (No.2060302).

and **will appear in the published article** if the submission is accepted. Please make sure it is accurate.<br><br>**Funded studies**<br>Enter a statement with the following details:<br>• Initials of the authors who received each award<br>• Grant numbers awarded to each author<br>• The full name of each funder<br>• URL of each funder website<br>• Did the sponsors or funders play any role in the study design, data collection and analysis, decision to publish, or preparation of the manuscript?<br><br>Did you receive funding for this work?"	
Please select the country of your main research funder (please select carefully as in some cases this is used in fee calculation).<br>as follow-up to "**Financial Disclosure**<br><br>Enter a financial disclosure statement that describes the sources of funding for the work included in this submission. Review the submission guidelines for detailed requirements. View published research articles from *PLOS ONE* for specific examples.<br><br>This statement is required for submission and **will appear in the published article** if the submission is accepted. Please make sure it is accurate.<br><br>**Funded studies**<br>Enter a statement with the following details:<br>• Initials of the authors who received each award<br>• Grant numbers awarded to each author<br>• The full name of each funder<br>• URL of each funder website<br>• Did the sponsors or funders play any role in the study design, data collection and analysis, decision to publish, or preparation of the manuscript?	CHINA - CN

Did you receive funding for this work?"	
**Competing Interests**<br><br>Use the instructions below to enter a competing interest statement for this submission. On behalf of all authors, disclose any competing interests that could be perceived to bias this work—acknowledging all financial support and any other relevant financial or non-financial competing interests.<br><br>This statement is required for submission and **will appear in the published article** if the submission is accepted. Please make sure it is accurate and that any funding sources listed in your Funding Information later in the submission form are also declared in your Financial Disclosure statement.<br><br>View published research articles from *PLOS ONE* for specific examples.<br><br>**NO authors have competing interests**<br><br>Enter: *The authors have declared that no competing interests exist.*<br><br>**Authors with competing interests**<br><br>Enter competing interest details beginning with this statement:<br><br>*I have read the journal's policy and the authors of this manuscript have the following competing interests: [insert competing interests here]*<br><br>* typeset	The authors have declared that no competing interests exist.
**Ethics Statement**<br><br>Enter an ethics statement for this submission. This statement is required if the study involved:	The treatment of animals during the experiment was in accordance with the animal ethics requirements stipulated in the "Regulations on the Administration of Laboratory Animals" of the State Science and Technology Commission and the "Beijing Laboratory Animal Welfare Ethical Review Guidelines" of Beijing. Approved by the Ethics Committee of Xiyuan Hospital, China Academy of Chinese Medical Sciences (№: 2021XLC002-2).

Powered by Editorial Manager® and ProduXion Manager® from Aries Systems Corporation

- Human participants
- Human specimens or tissue
- Vertebrate animals or cephalopods
- Vertebrate embryos or tissues
- Field research

Write "N/A" if the submission does not require an ethics statement.

General guidance is provided below. Consult the submission guidelines for detailed instructions. **Make sure that all information entered here is included in the Methods section of the manuscript.**

**Format for specific study types**<br><br>**Human Subject Research (involving human participants and/or tissue)**<br>• Give the name of the institutional review board or ethics committee that approved the study<br>• Include the approval number and/or a statement indicating approval of this research<br>• Indicate the form of consent obtained (written/oral) or the reason that consent was not obtained (e.g. the data were analyzed anonymously)<br><br>**Animal Research (involving vertebrate animals, embryos or tissues)**<br>• Provide the name of the Institutional Animal Care and Use Committee (IACUC) or other relevant ethics board that reviewed the study protocol, and indicate whether they approved this research or granted a formal waiver of ethical approval<br>• Include an approval number if one was obtained<br>• If the study involved *non-human primates*, add *additional details* about animal welfare and steps taken to ameliorate suffering<br>• If anesthesia, euthanasia, or any kind of animal sacrifice is part of the study, include briefly which substances and/or methods were applied<br><br>**Field Research**<br><br>Include the following details if this study involves the collection of plant, animal, or other materials from a natural setting:<br>• Field permit number<br>• Name of the institution or relevant body that granted permission	
**Data Availability**<br><br>Authors are required to make all data underlying the findings described fully available, without restriction, and from the time of publication. PLOS allows rare exceptions to address legal and ethical concerns. See the PLOS Data Policy and FAQ for detailed information.	Yes - all data are fully available without restriction

Powered by Editorial Manager® and ProduXion Manager® from Aries Systems Corporation

A Data Availability Statement describing where the data can be found is required at submission. Your answers to this question constitute the Data Availability Statement and **will be published in the article**, if accepted.<br><br>**Important:** Stating 'data available on request from the author' is not sufficient. If your data are only available upon request, select 'No' for the first question and explain your exceptional situation in the text box.<br><br>Do the authors confirm that all data underlying the findings described in their manuscript are fully available without restriction?	
**Describe where the data may be found in full sentences. If you are copying our sample text, replace any instances of XXX with the appropriate details.**<br><br>• If the data are **held or will be held in a public repository**, include URLs, accession numbers or DOIs. If this information will only be available after acceptance, indicate this by ticking the box below. For example: *All XXX files are available from the XXX database (accession number(s) XXX, XXX.).*<br>• If the data are all contained **within the manuscript and/or Supporting Information files**, enter the following: *All relevant data are within the manuscript and its Supporting Information files.*<br>• If neither of these applies but you are able to provide **details of access elsewhere**, with or without limitations, please do so. For example:<br><br>*Data cannot be shared publicly because of [XXX]. Data are available from the XXX Institutional Data Access / Ethics Committee (contact via XXX) for researchers who meet the criteria for access to confidential data.*<br><br>*The data underlying the results presented in the study are available from (include the name of the third party*	All relevant data are within the manuscript and its Supporting Information files.

and contact information or URL).<br>• This text is appropriate if the data are owned by a third party and authors do not have permission to share the data.<br><br>* typeset	
Additional data availability information:	

Powered by Editorial Manager® and ProduXion Manager® from Aries Systems Corporation

Manuscript Click here to access/download;Manuscript;manuscript.docx

Combined metabolomics and proteomics analysis of vascular cognitive impairment in hypertensive rats induced by endothelial injury

Xiao Zhang[1]， Surui Chang[1,2]， Jiangang Liu[1*,] Xiaoyuan Wang[1]

（1.Xiyuan Hospital, China Academy of Chinese Medical Sciences, National Center for Clinical Cardiovascular Disease of Traditional Chinese Medicine, Beijing, China; 2. Haikou Traditional Chinese Medicine Hospital, Haikou, Hainan, China）

Corresponding authors: E-mail: liujiangang2002@sina.com(JGL)

Abstract

Aim To study the molecular biological mechanism of vascular cognitive impairment model rats induced by hypertension combined with endothelial injury based on metabolomics and proteomics, and to find proteins or metabolites related to key metabolic pathways.

Methods SPF hypertensive rats (SHR) were used to damage the common carotid artery endothelium with microcurrent to induce vascular cognitive impairment model in rats (Model group), SHR rats (SHR group) and SPF normotensive Wistar-Kyoto rats (WKY) with the same genetic background were used as sham operation groups (no current stimulation after dissection), with 10 rats in each group. Morris water maze, PNT experiment and SPT experiment were used to detect the learning and memory ability of rats. TMT quantitative proteomics technology combined with liquid chromatography tandem mass spectrometry (LC-MS/MS) was used to detect the difference in metabolites and proteins in brain tissue of rats with vascular cognitive impairment.

Results From Day 1 to 5, compared with the WKY group, both the Model and SHR groups

exhibited shortened incubation periods and slower average swimming speeds, with statistical differences ($P<0.01$); On Day 6, the number of Model group crossings was significantly reduced ($P<0.05$), the time spent searching for the target quadrant was reduced ($P<0.05$), and the total distance traveled by the target quadrant was reduced ($P<0.05$). The pathological results showed that compared with the WKY group, the number of glial cells and neuronal cells in the brain hippocampus of rats in the SHR group and the Model group was reduced, the cell bodies were relatively loosely arranged, and Nissl body staining was lighter. The morphology of some hippocampal neurons in the Model group changes from granular to powdery, The morphology of some hippocampal neurons in the Model group changed from granular to powdery, with nuclear consolidation, accompanied by cellular edema and necrosis. The metabolomics results showed that there were 437 significantly different metabolites between the Model group and the WKY group, 128 (+) and 309 (-); there were 449 significantly different metabolites between the Model group and the SHR group, 119 (+), 330(-). The differential metabolites in each group were mainly concentrated in metabolic pathways such as alanine, aspartate and glutamate metabolism, arginine and proline metabolism, and amino acid biosynthesis. The proteomics results showed that compared with the WKY group, there were 141 differentially expressed proteins in the Model group, 55 (+), and 86 (-). Pathways with higher connectivity in the action network include "glycine, serine and threonine metabolism", "phenylalanine metabolism", etc.; compared with the SHR group, there were 28 differentially expressed proteins in the Model group and 15 (+). 13 species (-). The significantly enriched pathways are "focal adhesion" and "relaxin signaling pathway".

Conclusion Rats with hypertension combined with endothelial injury have behavioral disorders, which are similar to the causes and signs of clinical vascular cognitive impairment; the biological mechanisms manifested in the model involve amino acid metabolism disorders, relaxin signaling

pathway and impaired focal adhesion function.

Keywords vascular cognitive impairment; vascular dementia; hypertensive rats; hypertension; endothelial dysfunction; metabolomics; proteomics

Background

Vascular cognitive impairment (VCI) is caused by the dysfunction of the neurovascular unit and cerebral blood flow regulation disorders, involving damage to the endothelial cells of the vascular wall and the glial cells outside the vascular wall, oxidative stress and immune inflammatory response, which ultimately leads to neuronal damage, which is the key factor in the dysfunction of the neurovascular unit[1]. Clinical manifestations include communication disorders, memory loss, distraction, personality changes, etc. Vascular dementia (VD) is its severe stage, which refers to dementia caused by a series of cerebrovascular factors (ischemia, hemorrhage or acute and chronic hypoxic cerebrovascular disease, etc.) that cause brain tissue damage. Epidemiology shows that the number of dementia patients doubles approximately every five years. VD accounts for at least 20% to 40% of all dementia diagnoses, and the prevalence of VD in individuals aged 50 years and above is about 116/10,000 people [2,3]. It is currently believed that one of the most important types of pathophysiological factors leading to vascular dementia is ischemic and hypoxic hypoperfusion[4]. Due to hypertension and arteriosclerosis, the important parts of the cerebral cortex involved in cognitive function and the brain tissue that is

more sensitive to ischemia and hypoxia are in a state of ischemic hypoperfusion for a long time, which causes delayed necrosis of neurons in these parts and gradually leads to cognitive dysfunction. At present, most of the vascular cognitive impairment models are made by vascular occlusion and vascular embolization [5]. Although the conditions of brain hypoperfusion can lead to dementia-like cognitive dysfunction, they are still quite different from the causes of vascular dementia in clinical patients. Hypertension is a common factor in VCI [6]. When cerebral blood vessels are exposed to high pulsatile pressure and flow, microvascular damage occurs, resulting in white matter damage and cortical detachment, which leads to the occurrence of VCI and VD [7].

We used hypertensive rats combined with microcurrent to injure their carotid endothelium to cause thrombosis, resulting in repeated cerebral ischemia and hypoxia-induced vascular cognitive impairment models. Metabolomics and proteomics are used to focus on the changes in small molecule metabolites and protein composition in the body, hoping to fully reflect the metabolic network of VCI rats and reveal the complex regulatory network of protein levels during the pathogenesis of VCI. Through joint analysis, the interaction and regulatory relationship between proteins and metabolites can be found. Therefore, in this study, we explored the formation mechanism of hypertension combined with carotid endothelial injury to vascular cognitive impairment model rats based on metabolomic and proteomic technologies, proposed a molecular biology change mechanism model, and jointly analyzed the expression data of differentially expressed proteins and metabolites to find out the differentially expressed proteins

and metabolites that have synchronous rule of change, and then performed personalized analysis by combining with correlation coefficient matrix heatmap, correlation analysis clustering heatmap and correlation coefficient regulatory network diagram for personalized analysis. This provides a data basis for subsequent in-depth experiments and analysis, and explores possible intervention targets or signal pathways for VCI and VD.

1. Materials and methods

1.1 Experimental animals

Spontaneously hypertensive rats (SHR), Specific pathogen free (SPF), male, 13-14 weeks old, 15; Kyoto Wistar rats (Wistar-Kyoto, WKY) of the same genetic background, male, 13-14 weeks old, 15 rats; all were purchased from Beijing Viton Lihua Laboratory Animal Technology Co. Barrier-grade animals were kept indoors and fed with complete nutritional feed at regular intervals. The maintenance feed was purchased from Beijing Keao Xieli Feed Co., Ltd., production certificate number: Beijing Feed Certificate (2017) 06054. The room temperature was maintained at (22-25)°C, the humidity was maintained at (50-70)%, and the light and dark time was half (12 h). Rats were free to eat and drink water. The experiment was conducted after 1 week of adaptation.

1.2 Experimental instruments

QTRAP5500 mass spectrometer, produced by Shanghai Aibocaisi Analytical Instrument Trading Co., Ltd.; Nexera X2 LC-30AD high performance liquid chromatograph, produced by Shimadzu (Shanghai) Laboratory Equipment Co., Ltd.; Waters Acquity UPLC HSS T3 column, produced by Waters Technology (Shanghai) Co., Ltd.

QE HF-X high-resolution orbitrap liquid-mass spectrometer, provided by Thermo Fisher Scientific (China) Co., Ltd.; EASY-nLC 1200 chromatograph, provided by Thermo Fisher

Scientific (China) Co., Ltd.; 89870 reversed-phase HPLC column, provided by Thermo Fisher Scientific (China) Co., Ltd.; SC601 EASY-Column™ capillary HPLC column, provided by Thermo Fisher Scientific (China) Co., Ltd.; MultiSkan FC MultiskanSkyHigh full-wavelength microplate reader, provided by Thermo Fisher Scientific (China) Co., Ltd.; Pierce™ High pH Reversed-Phase Peptide Fractionation Kit, provided by Thermo Fisher Scientific (China) Co., Ltd.; TMTpro™ 16plex labeling reagent (TMTpro plex kit), provided by Thermo Fisher Scientific (China) Co., Ltd.

1.3 Preparation and grouping of vascular dementia model induced by microcurrent stimulation of bilateral common carotid arteries in SHR

Prepare rats with vascular dementia model induced by microcurrent stimulation of bilateral common carotid arteries. Preoperative preparation: Rats were fasted for 12 h, and routine aseptic operation was performed. 2% sodium pentobarbital (0.3 ml/100 g body weight) was injected intraperitoneally for anesthesia. After anesthesia, the rats were fixed in supine position, the neck was disinfected with skin and iodine, and the skin was cut longitudinally along the middle of the neck. The skin and muscle layer of the neck were separated by 1.5-2.0 cm, the bilateral common carotid arteries were exposed, and the common carotid arteries and their accompanying nerves were peeled off to the head and heart sides. Two sections of surgical line were clamped and passed through the bottom of the common carotid artery and then arranged in parallel. The two ends of the surgical line were placed on the left and right sides of the blood vessels respectively, and the surgical line was lifted to place the gasket under the blood vessel. Turn on the power, turn the timing and stimulation switches to the ready position, and prepare the stimulation electrodes and temperature probe. Then connect the power plug to the 220V power supply. Before each operation, clear the digital tube numbers, adjust the zero adjustment knob to control the potential difference (voltage) at 80mV, and adjust the current timing switch to make the

microcurrent (0.3A) stimulation timer to 3min. Then place the electrodes on the bilateral common carotid arteries of SHR rats, and shock the common carotid arteries of rats at the set power and time until the instrument automatically alarms to indicate thrombosis, as shown in Figure 1.

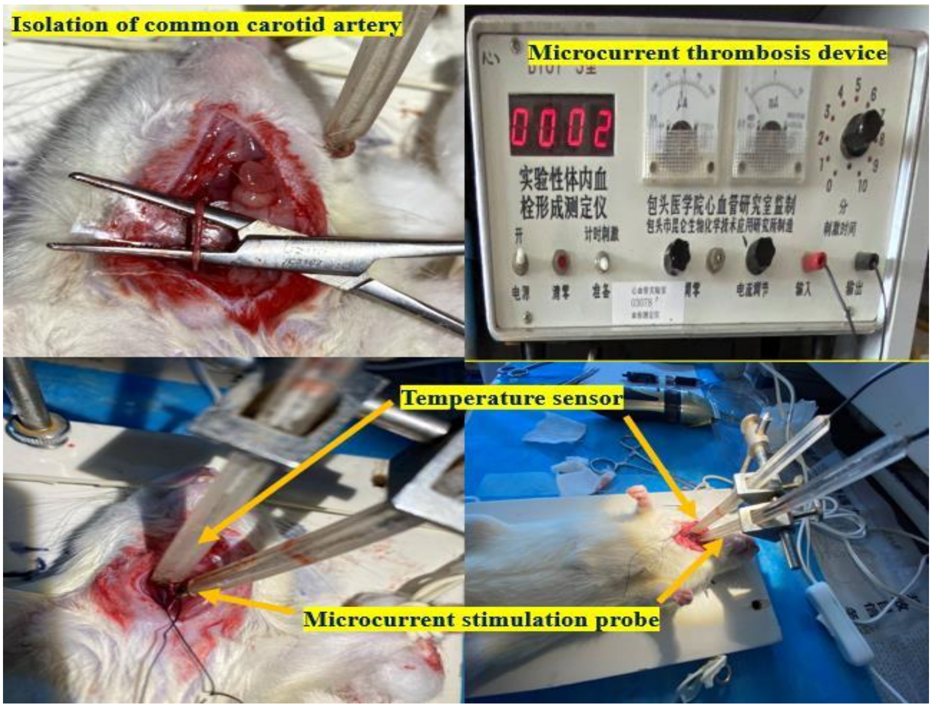

Figure 1. Experimental microcurrent stimulation of in vivo thrombosis instrument operation process (The common carotid artery of rats was isolated and stimulated by microcurrent. The temperature sensor was hooked on the distal segment of the artery and microcurrent stimulated thrombosis in vivo.)

In the WKY group, bilateral common carotid arteries were bluntly separated, and only threading was performed without electric stimulation; after the operation, the gasket and surgical thread were removed from under the blood vessels and sutured layer by layer. After the operation, pay attention to keeping warm, closely observe the wound healing after waking up, and inject penicillin (20IU/kg) intramuscularly for 3 consecutive days to prevent infection. Morris water

maze experiment was performed 1 week after modeling, and rats with behavioral cognitive impairment were screened as the model group by comparison with the WKY group. Rats with cognitive impairment were screened as the SHR+vascular injury composite model group (Model group), and SHR rats (SHR group) and normal blood pressure Wistar-Kyoto rats (WKY) with the same genetic background were selected as the sham operation group (WKY group), with 10 rats in each group. The treatment of animals during the experiment was in accordance with the animal ethics requirements stipulated in the "Regulations on the Administration of Laboratory Animals" of the State Science and Technology Commission and the "Beijing Laboratory Animal Welfare Ethical Review Guidelines" of Beijing. Approved by the Ethics Committee of Xiyuan Hospital, China Academy of Chinese Medical Sciences (№: 2021XLC002-2).

1.4 Rat behavioral test

The Morris water maze (MWM) was used to evaluate the spatial learning and memory function of the model rats. The equipment includes a circular pool (about 50 cm high and about 120 cm in diameter), an automatic camera, and a computer video analysis system. The pool is divided into four quadrants in a clockwise direction, and a platform is placed in the center of the first quadrant. Clean water is poured about 1 cm above the platform, and ink is added to the water to hide the platform. The water temperature is maintained at (21±2)°C. The indoor environment and light source are kept quiet during the experiment. The rats were placed in the MWM laboratory 1 day before the experiment to adapt to feeding. During the experiment, the rats were placed in the water with their heads down and their backs facing the pool wall. Each round lasted 60 seconds, and the test interval was no less than 30 minutes to ensure adequate rest. A total of 6 days. The first 5 days were the Place navigation test (PNT) to evaluate the spatial learning ability of the rats. The Spatial probe test (SPT) on the 6th day was used to evaluate the memory ability of the rats.

PNT experiment: Rats were randomly placed into the water at four locations: east (E), north (N), northwest (NW), and southeast (SE) during a fixed period of time. The escape latency (s), swimming distance (cm), and swimming speed (cm/s) of the rats were observed. If the rats did not get on the platform for more than 60 seconds, they were guided to the platform and stayed there for 10 seconds.

SPT experiment: Remove the platform and enter the water at the northwest (NW) point. Record the number of times the rats cross the original platform (times), the activity time in the target quadrant (s) and the distance (cm) to evaluate the rats' learning and memory ability.

1.5 Observation of pathological structure of cortical and hippocampal synapses

1.5.1 HE staining experiment

Immerse the rat brain tissue in 10% neutral formaldehyde solution for fixation, followed by embedding, deparaffinization, and gradual ethanol dehydration. Stain with 1% hematoxylin and eosin, and mount with neutral gum for observation under an optical microscope. Use an HE staining kit to stain and capture images at ×200 and ×400 magnifications in the hippocampal CA1 and CA3 regions, selecting neurons with uniform staining and relatively intact structure to showcase the pathological morphology of the brain tissue.

1.5.2 Nissl staining experiment

Under an optical microscope, observe the Nissl bodies in the neurons of the CA1 and CA3 regions of the hippocampus at ×200 and ×400 magnification, observe the neuronal cell structure, and understand the damage of the neurons. Toluidine blue method operation steps: ① Fresh tissue is fixed in 10% formaldehyde solution and then dehydrated and embedded in the conventional method; ② Paraffin sections with a thickness of 5um are routinely dewaxed to water; ③ Use Nissl staining solution to place in a 50~60℃ incubator for 20 to 40 minutes; ④ Wash with distilled water and then quickly differentiate with 95% ethanol, dehydrate with

anhydrous ethanol and then make xylene transparent, and finally seal with neutral gum.

1.6 Metabolomics detection

Chromatographic conditions: During the entire analysis process, the sample was placed in an autosampler at 4°C, and the sample was separated using a Shimadzu Nexera X2 LC-30AD ultra-high performance liquid chromatography system (UHPLC) using a T3 chromatographic column. Mass spectrometry conditions: Each sample was detected using electrospray ionization (ESI) in positive ion (+) and negative ion (-) modes. The samples were separated by UPLC and analyzed by mass spectrometry on a 5500 QTRAP mass spectrometer, using a HESI source for ionization. Use MRM mode to detect the ion transition to be measured. According to the variable weight value (Variable Importance for the Projection, VIP) obtained by the OPLS-DA model, the influence intensity and explanatory ability of the expression pattern of each metabolite on the classification and discrimination of each group of samples were measured. In this experiment, VIP>1 was used as the screening criterion. Preliminarily screen out the differences between each group. Univariate statistical analysis was further used to verify whether the metabolites had significant differences. Metabolites with both multidimensional statistical analysis VIP>1 and univariate statistical analysis $P<0.05$ were selected as metabolites with significant differences.

1.7 Protein testing

TMT proteomics methods were used to detect and analyze proteins in brain tissue. For each sample, appropriate amounts of peptide fragments were taken for chromatographic separation using the nanoflow rate EasyLC1200 chromatography system. After peptide separation, DDA (data-dependent acquisition) mass spectrometry was performed using a Q-Exactive HF-X mass spectrometer. The analysis time is 60 minutes, detection mode: positive ion, precursor ion scanning range: 300-1800m/z, primary mass spectrometry resolution: 60000@m/z200, AGC target: 3e6, primary Maximum IT: 50ms. Secondary mass spectrometry analysis of peptides was

collected according to the following method: triggering the acquisition of 20 highest intensity precursor ion secondary mass spectra (MS2scan) after each full scan, secondary mass spectrometry resolution: 15000@m/z200, AGC target: 1e5, Level 2 Maximum IT: 50ms, MS2ActivationType: HCD, Isolation window: 1.6m/z, Normalized collision energy: 32. Proteome and Discoverer software were used for qualitative and quantitative calculations of TMT-labeled proteomic data. In the significant difference analysis of quantitative results, proteins that meet the screening criteria of expression difference greater than 1.2 times (up and down regulation) and *P<0.05* in the pairwise comparison groups are regarded as significantly differentially expressed proteins.

1.8 Biological analysis of proteomics and metabolomics

The biological analysis of metabolomics uses multivariate statistical analysis methods to "simplify and reduce the dimensionality" of high-dimensional and complex data, and then summarize and summarize the metabolic profile characteristics of the research objects. Calculating the Variable Importance for the Projection (VIP) of OPLS-DA can measure the importance of differential metabolites. Univariate statistical analysis, using FC>1.5 or FC<0.667, and P<0.05 as the standard to screen potential marker metabolites, draw a volcano plot, each dot represents a metabolite, gray indicates that the difference is not significant, and red dots Expression is significantly up-regulated (+), blue dot expression is significantly down-regulated (-). Expression analysis of differential metabolites combines multivariate statistical analysis with univariate statistical analysis to screen metabolites that meet VIP>1.0 and P<0.05 as differential metabolites. Functional analysis of differential metabolites, and classification statistics of differential metabolite data of each comparison group. For KEGG pathway analysis of differential metabolites, P < 0.05 was used as the screening criterion to construct a pathway network enriched by differential metabolites and draw the "KEGG pathway PPI".

Proteomics uses Microsoft Excel, Perseus software and R software to analyze bioinformatics data. Combined with the GO database, KEGG database, and UniProtKB database, the full protein function, differential protein expression between groups, and differential protein expression among multiple groups were analyzed. Use STRING database and cytoscape software to construct the "Interaction Network (PPI)". Comprehensive interaction network analysis integrates the "protein-protein" interaction relationships and "pathway-protein" relationships of the STRING database into a network, which is conducive to in-depth research on protein functions. The study draws a diagram of "Top5 significantly enriched pathways and the protein/protein coding genes and protein interaction network in this pathway." In all the above analyses, "+" is used to indicate significant up-regulation of proteins, genes, and pathways, and "-" is used to indicate significant down-regulation.

1.9 Statistical analysis and processing

The obtained data were expressed as $\bar{x}\pm s$. The data were tested for normal distribution and homogeneity of variance. The homogeneity of variance was tested using one-way ANOVA. Multiple comparisons between groups were performed using the LSD test method. Dunnett's T3 analysis method was used when the variance was unequal. Bioinformatics data were analyzed using Microsoft Excel, Perseus software, and R software. SPSS 27.0 software was used to analyze the data. $P<0.05$ was considered statistically significant.

2. Results

2.1 Behavioral comparison between rats in each group

The results showed that from D1 to D5, compared with the WKY group, the latency period of the Model group and the SHR group was shortened, and the average swimming speed was increased, with statistical differences ($P<0.01$). The escape latency of the three groups showed a decreasing trend, see Figure 1. A. D6 removes the platform and begins space exploration testing.

Place the rat into the pool from the opposite side of the original platform quadrant. Record the time it takes to search the target quadrant (Quadrant I) and the number of times it enters this quadrant, as a detection indicator of spatial memory. Compared with the WKY group, the number of platform crossings in the Model group was significantly reduced (*P<0.05*), the time spent searching for the target quadrant was reduced (*P<0.05*), and the total distance traveled by the target quadrant was reduced (*P<0.05*, Figure 2).

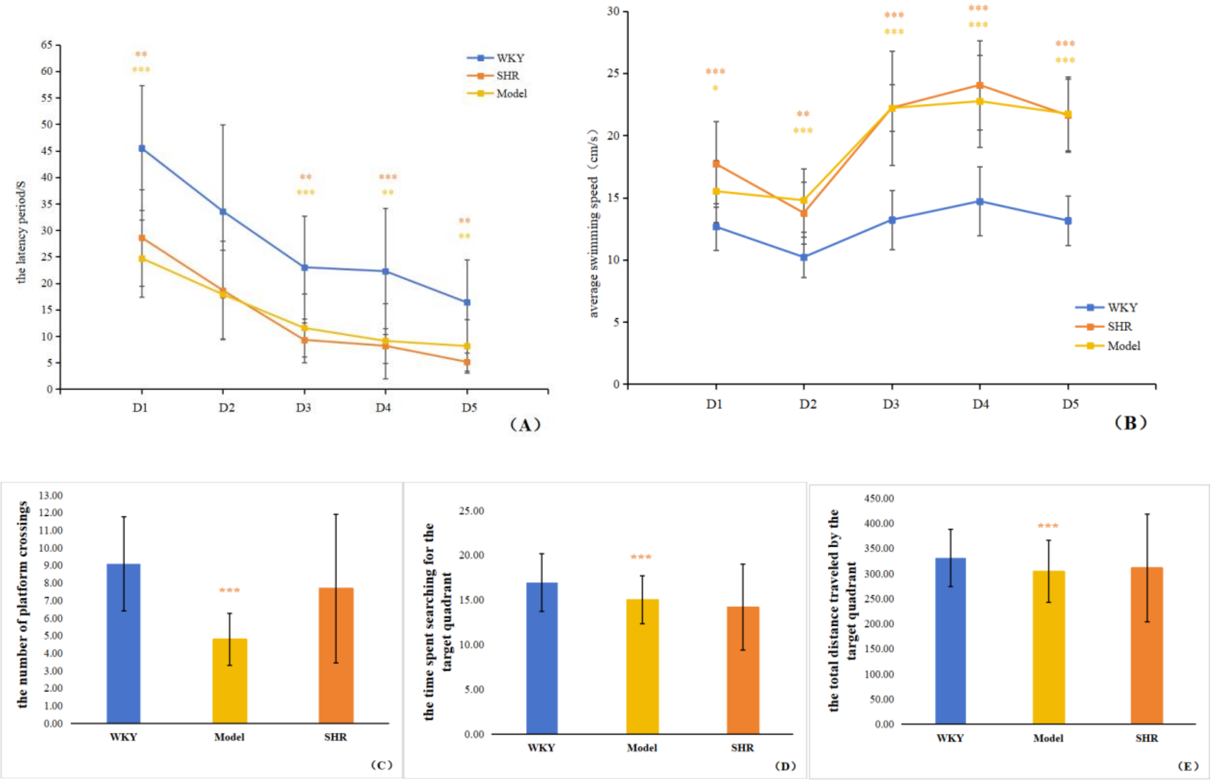

Figure 2. (A) Escape latency; (B) Average swimming speed; (C) Number of times rats crossed the platform; (D) Time spent in the target quadrant of rats; (E) Distance traveled in the target quadrant of rats. **P<0.05* compared with the WKY group; ***P<0.01* compared with the WKY group; ****P<0.001* compared with the WKY group

2.2 Pathological staining

2.2.1 Observation of pathological changes in the hippocampus of rats in each group by HE staining

The results showed that the hippocampus of rats in the WKY group had normal tissue structure, with dense and orderly arrangement of neurons, uniform staining, full and regular morphology, and normal nuclear staining. Compared with the WKY group, the number of neurons in the hippocampus of rats in the SHR group and the Model group was relatively reduced, with deformation and shrinkage of glial cell nuclei, and relatively loose arrangement of glial cells (Figure 3).

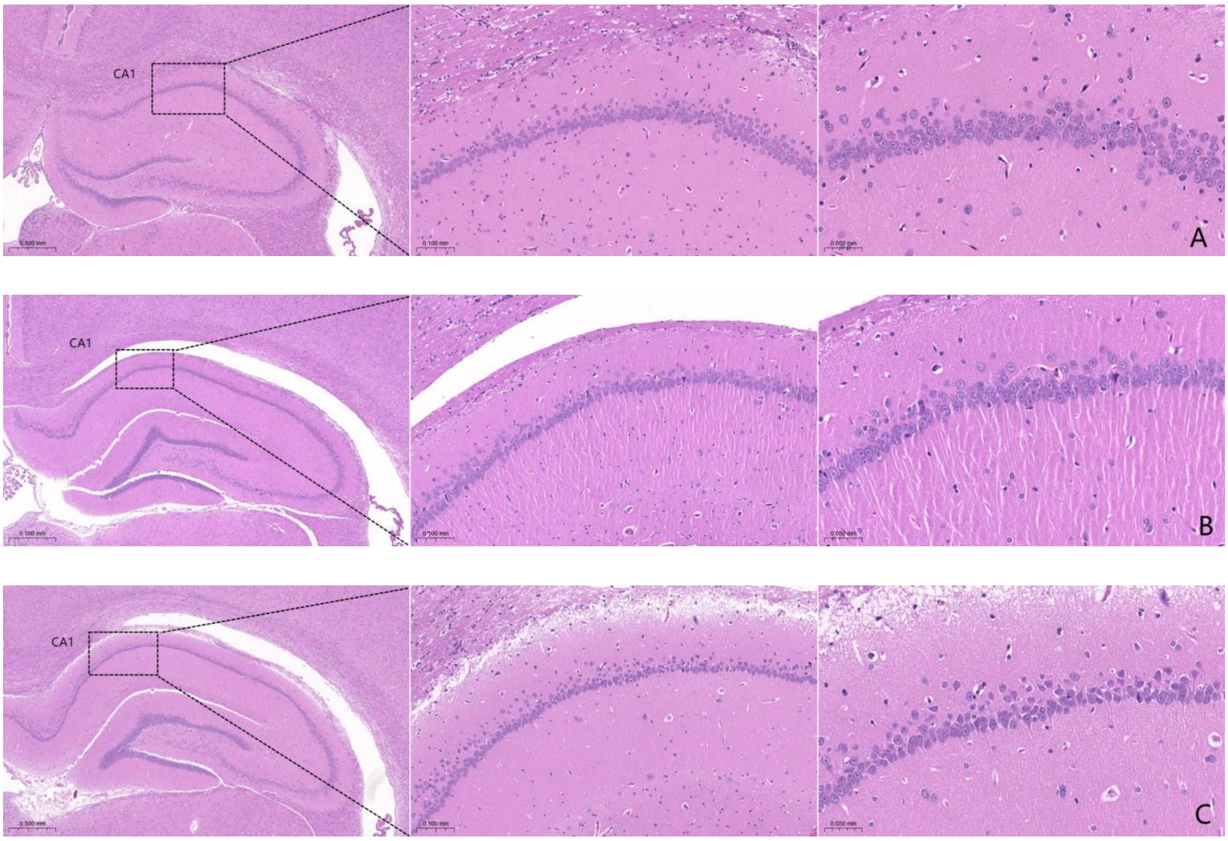

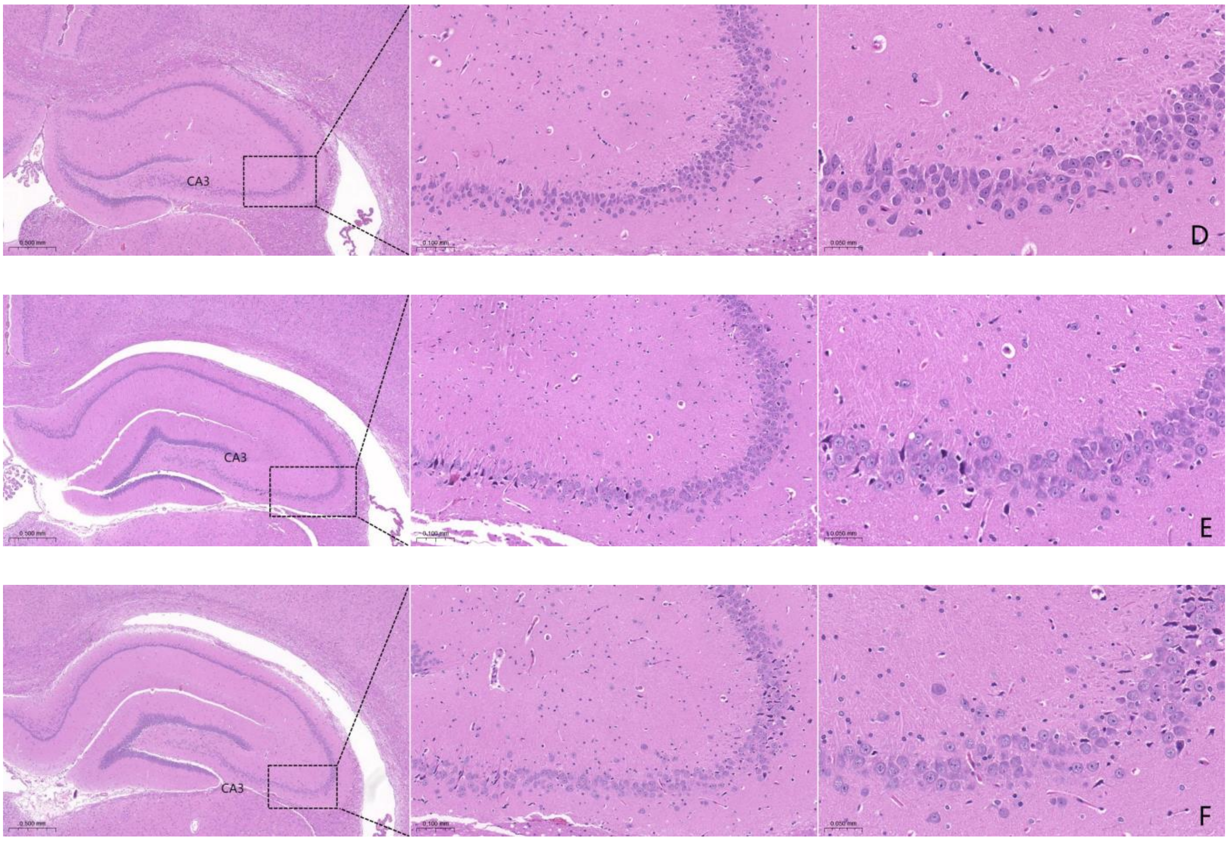

Figure 3. Pathological observation of CA1 and CA3 regions of the hippocampus in each group (A. CA1 region of the WKY group; B. CA1 region of the SHR group; C. CA1 region of the Model group; D. CA3 region of the WKY group; E. CA3 region of the SHR group; F. CA3 region of the Model group; HE staining, ×200, bar: 100um; ×400, bar: 50um)

2.2.2 Observation of pathological changes of Nissl staining in the hippocampus of rats in each group

The results showed that neurons in the CA1 and CA3 regions of the hippocampus in the WKY group were arranged regularly, the Nissl bodies and cell nuclei of the neurons were blue-purple, and the Nissl bodies were granular in shape and abundant in number. The hippocampal neurons in the SHR and Model groups were arranged relatively loosely, and the Nissl bodies were stained lightly. The morphology of some hippocampal neurons in the Model group changed from granular to powdery, and the nuclei were condensed, accompanied by cell edema and

necrosis (Figure 4).

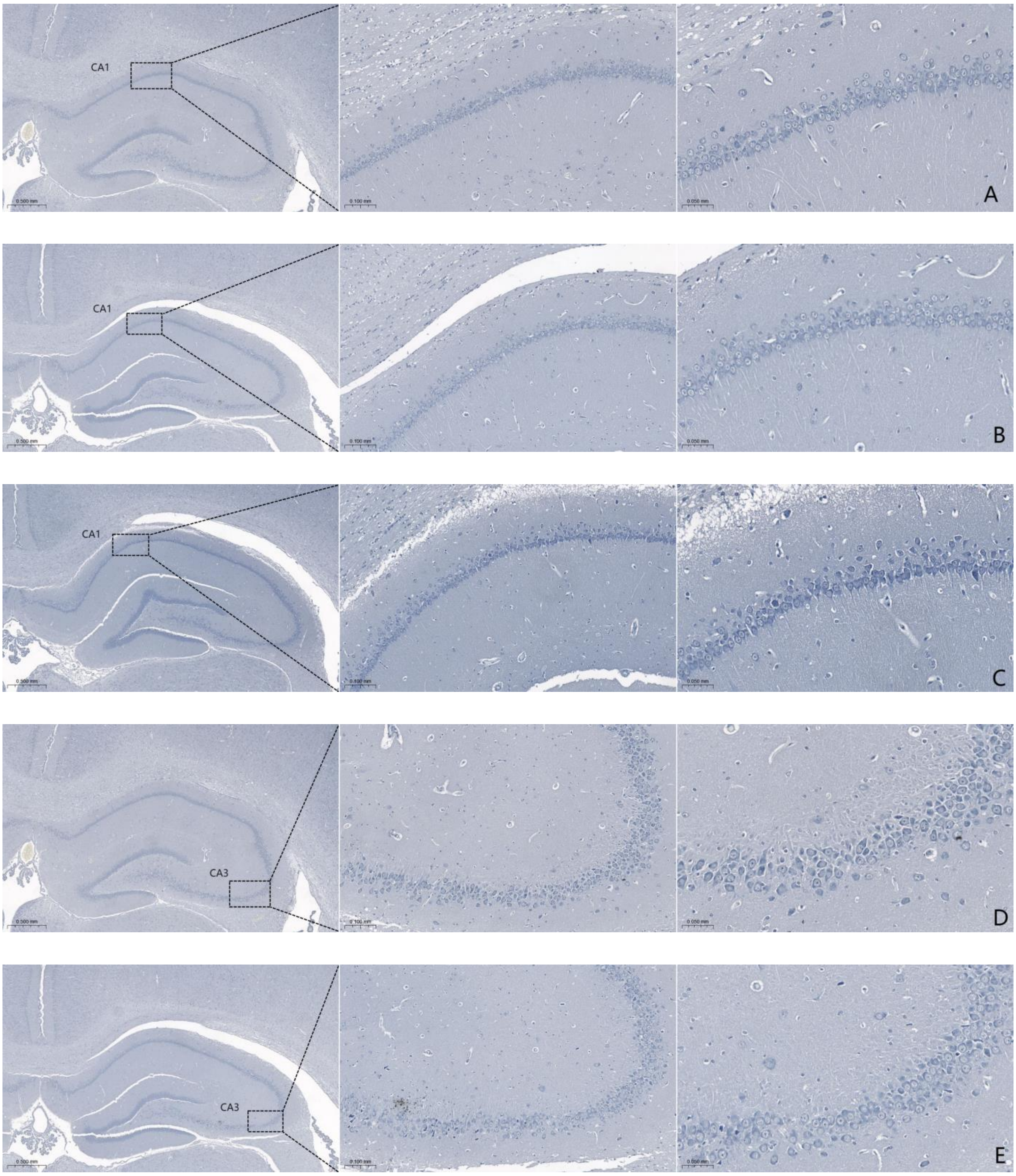

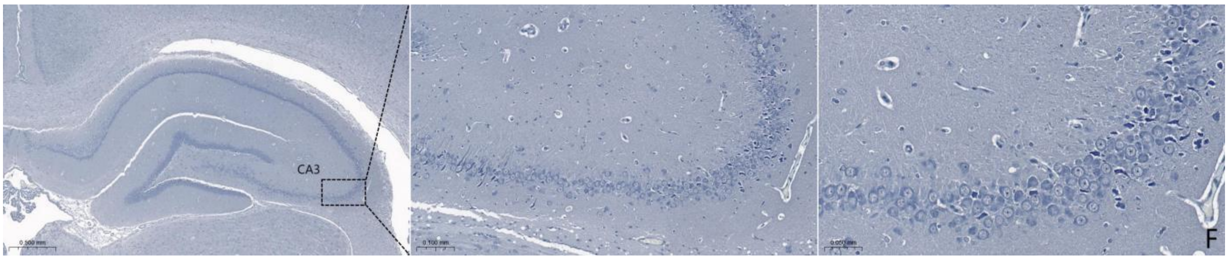

Figure 4. Pathological observation of CA1 and CA3 regions of the hippocampus in each group (A. CA1 region of the WKY group; B. CA1 region of the SHR group; C. CA1 region of the Model group; D. CA3 region of the WKY group; E. CA3 region of the SHR group; F. CA3 region of the Model group; Nissl staining, ×200, bar: 100um; ×400, bar: 50um)

2.3 Comparison of differential metabolites in rat brain tissue in each group

2.3.1 Expression and functional analysis of differential metabolites between Model group and WKY group

Compared with Model and WKY, there were 437 significantly different metabolites, 128 (+) and 309 (-). The top 50 differential metabolites are all expressed (-) and clustered into 12 categories: phosphatidyl acid (n=17), amino acids, peptides and analogs (n=13), amines, choline, and other organic nitrogen compounds (n=3), glycation end products (n=3), nucleosides, nucleotides and analogs (n=4), vitamins and their derivatives (n=2), sphingolipids (n=1), organic Acids and their derivatives (n=1), indoles and other heterocyclic compounds (n=2), fatty acids (n=2), flavonoids, benzene and substituted derivatives (n=1), acyl meat Base (n=1). See Table 1 and Figure 5 A~B. Analyze the data flow direction of differential metabolites and KEGG pathways. The Top15 pathways are shown in Table 1 and Figure 5C.

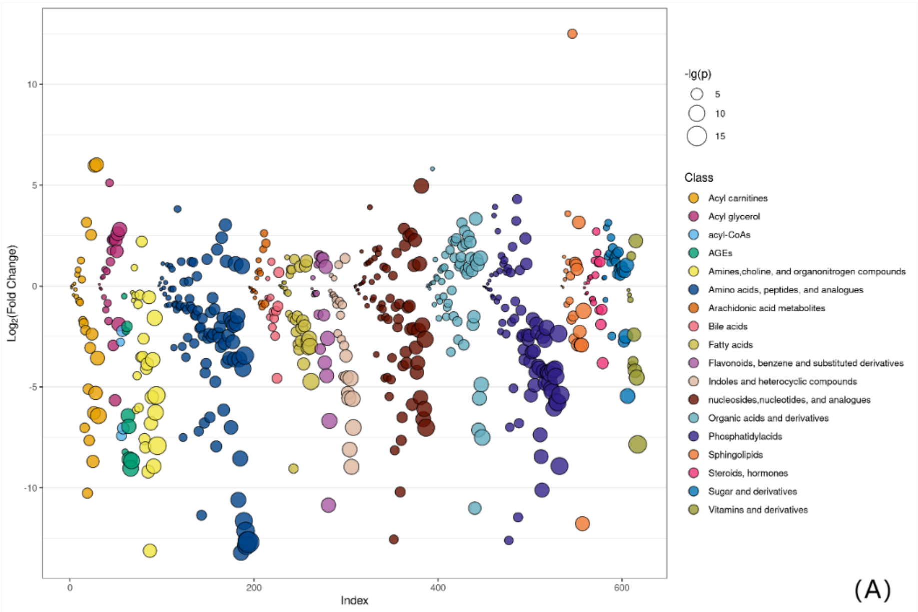
-lg(p)
5
10
15
Class
Acyl carnitines
Acyl glycerol
acyl-CoAs
AGEs
Amines,choline, and organonitrogen compounds
Amino acids, peptides, and analogues
Arachidonic acid metabolites
Bile acids
Fatty acids
Flavonoids, benzene and substituted derivatives
Indoles and heterocyclic compounds
nucleosides,nucleotides, and analogues
Organic acids and derivatives
Phosphatidylacids
Sphingolipids
Steroids, hormones
Sugar and derivatives
Vitamins and derivatives
Log2(Fold Change)
Index
(A)

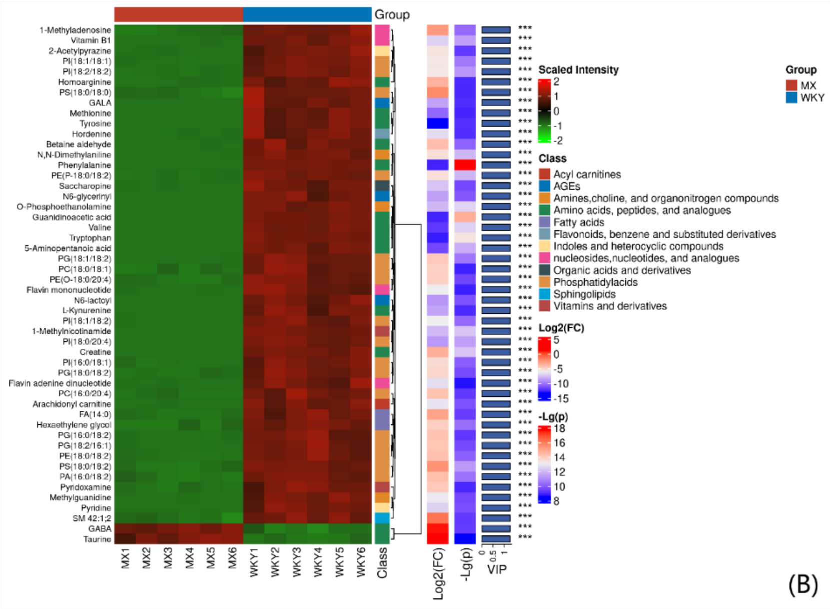
Group
1-Methyladenosine
Vitamin B1
2-Acetylpyrazine
PI(18:1/18:1)
PI(18:2/18:2)
Homoarginine
PS(18:0/18:0)
GALA
Methionine
Tyrosine
Hordenine
Betaine aldehyde
N,N-Dimethylaniline
Phenylalanine
PE(P-18:0/18:2)
Saccharopine
N6-glycerinyl
O-Phosphoethanolamine
Guanidinoacetic acid
Valine
Tryptophan
5-Aminopentanoic acid
PG(18:1/18:2)
PC(18:0/18:1)
PE(O-18:0/20:4)
Flavin mononucleotide
N6-lactoyl
L-Kynurenine
PI(18:1/18:2)
1-Methylnicotinamide
PI(18:0/20:4)
Creatine
PI(16:0/18:1)
PG(18:0/18:2)
Flavin adenine dinucleotide
PC(16:0/20:4)
Arachidonyl carnitine
FA(14:0)
Hexaethylene glycol
PG(16:0/18:2)
PG(18:2/16:1)
PE(18:0/18:2)
PS(18:0/18:2)
PA(16:0/18:2)
Pyridoxamine
Methylguanidine
Pyridine
SM 42:1;2
GABA
Taurine
MX1
MX2
MX3
MX4
MX5
MX6
WKY1
WKY2
WKY3
WKY4
WKY5
WKY6
Class
Log2(FC)
-Lg(p)
VIP
Scaled Intensity
Class
Acyl carnitines
AGEs
Amines,choline, and organonitrogen compounds
Amino acids, peptides, and analogues
Fatty acids
Flavonoids, benzene and substituted derivatives
Indoles and heterocyclic compounds
nucleosides,nucleotides, and analogues
Organic acids and derivatives
Phosphatidylacids
Sphingolipids
Vitamins and derivatives
Log2(FC)
-Lg(p)
Group
MX
WKY
(B)

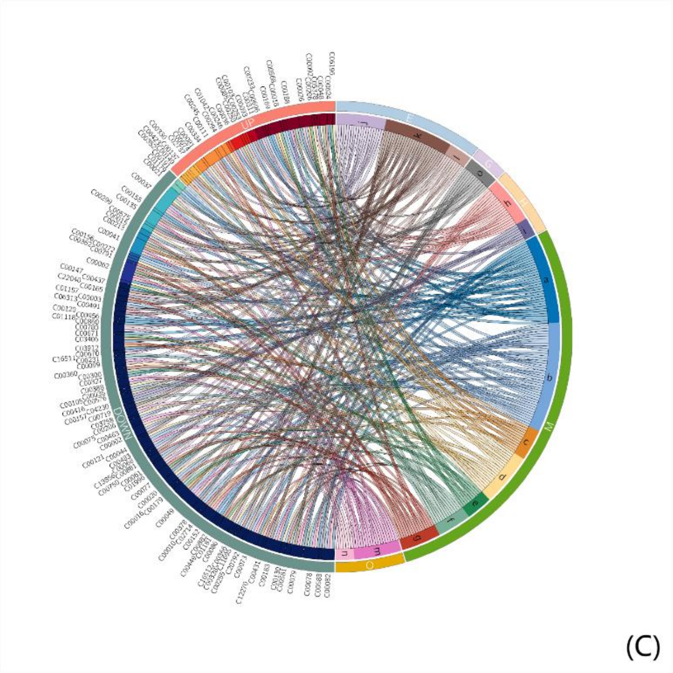

Figure 5. (A) FC and VIP bubble chart of Model.vs.WKY comparison group; (B) Hierarchical clustering results of differential metabolites in Model.vs.WKY comparison group (only the top 50 VIP values are plotted); (C) Chord diagram of enriched pathways of top 15 differential metabolites in Model.vs.WKY comparison group (Level 1 pathway classification: Metabolism (M), Genetic Information Processing (G), (Environmental Information Processing (E), Cellular Processes (C), Organic Systems (O), Human Diseases (H), Drug Development (D))

Table 1. Model vs. WKY group KEGG pathways (Top 15)

Description	pvalue	Count	UP	DOWN
ABC transporters	3.10652E-10	26	6	20
Alanine, aspartate and glutamate metabolism	9.95371E-10	12	6	6
Aminoacyl-tRNA biosynthesis	2.54628E-06	12	1	11
Arginine and proline metabolism	1.67072E-06	15	2	13
Arginine biosynthesis	2.23768E-08	10	3	7

Biosynthesis of amino acids	5.71287E-18	34	10	24
Biosynthesis of cofactors	2.94919E-10	42	11	31
cAMP signaling pathway	8.09722E-07	9	1	8
Central carbon metabolism in cancer	3.0881E-15	18	7	11
Choline metabolism in cancer	1.08741E-07	7	1	6
Glycine, serine and threonine metabolism	2.13186E-07	13	4	9
Neuroactive ligand-receptor interaction	3.17544E-13	19	3	16
Protein digestion and absorption	5.32119E-13	18	2	16
Purine metabolism	5.42789E-09	20	7	13
Synaptic vesicle cycle	2.50875E-07	7	1	6

2.3.2 Expression and functional analysis of differential metabolites between Model group and SHR group

Model and SHR ratio, 449 significantly different metabolites, 119 (+), 330 (-). The Top 50 differential metabolites are all expressed (-) and clustered into 11 categories: phosphatidyl acid (n=17), amino acids, peptides and analogs (n=8), nucleosides, nucleotides and analogs (n= 6), indoles and other heterocyclic compounds (n=5), amines, choline, and other organic nitrogen compounds (n=3), fatty acids (n=3), glycation end products (n=2), flavonoids, benzene and substituted derivatives (n=2), sphingolipids (n=2), vitamins and their derivatives (n=1), organic acids and their derivatives (n=1). See Table 2 and Figure 6 A~B. Analyze the data flow direction of differential metabolites and KEGG pathways. The Top15 pathways are shown in Table 2 and Figure 6 C.

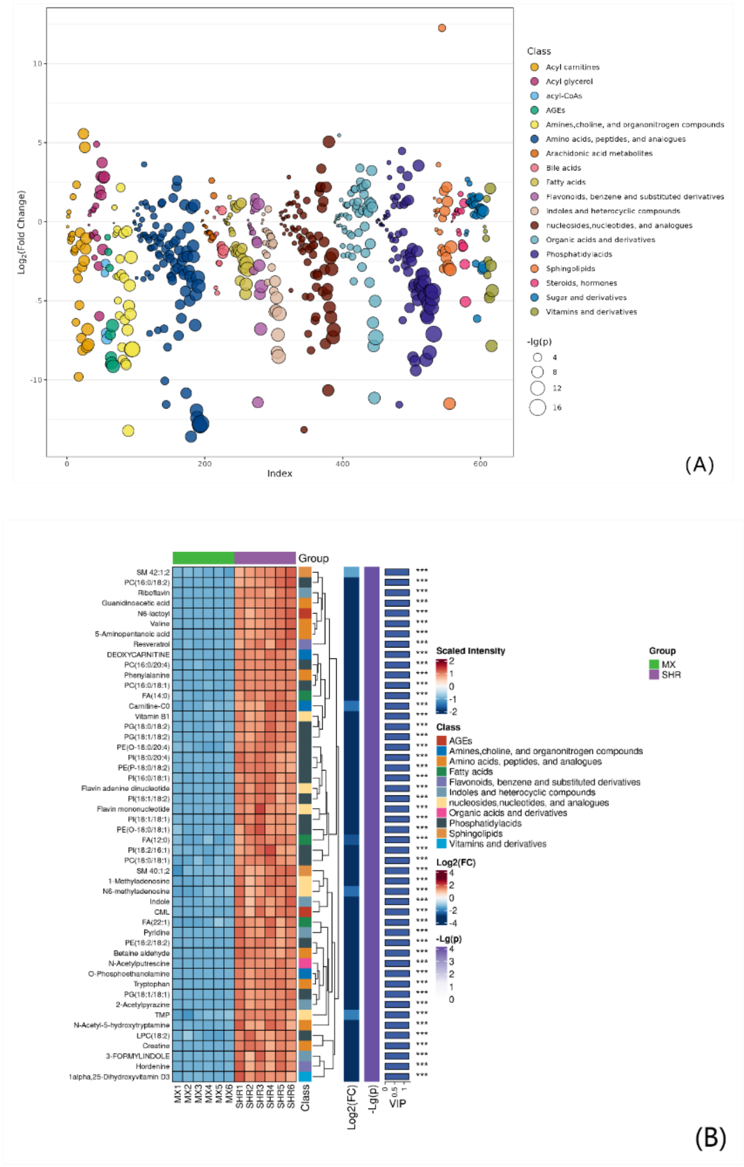
(A)
Log2(Fold Change)
Index
Class
Acyl carnitines
Acyl glycerol
acyl-CoAs
AGEs
Amines,choline, and organonitrogen compounds
Amino acids, peptides, and analogues
Arachidonic acid metabolites
Bile acids
Fatty acids
Flavonoids, benzene and substituted derivatives
Indoles and heterocyclic compounds
nucleosides,nucleotides, and analogues
Organic acids and derivatives
Phosphatidylacids
Sphingolipids
Steroids, hormones
Sugar and derivatives
Vitamins and derivatives
-lg(p)
4
8
12
16
(B)
Group
SM 42:1;2
PC(16:0/18:2)
Riboflavin
Guanidinoacetic acid
N6-lactoyl
Valine
5-Aminopentanoic acid
Resveratrol
DEOXYCARNITINE
PC(16:0/20:4)
Phenylalanine
PC(16:0/18:1)
FA(14:0)
Carnitine-C0
Vitamin B1
PG(18:0/18:2)
PG(18:1/18:2)
PE(O-18:0/20:4)
PI(18:0/20:4)
PE(P-18:0/18:2)
PI(16:0/18:1)
Flavin adenine dinucleotide
PI(18:1/18:2)
Flavin mononucleotide
PI(18:1/18:1)
PE(O-18:0/18:1)
FA(12:0)
PI(18:2/16:1)
PC(18:0/18:1)
SM 40:1;2
1-Methyladenosine
N6-methyladenosine
Indole
CML
FA(22:1)
Pyridine
PE(18:2/18:2)
Betaine aldehyde
N-Acetylputrescine
O-Phosphoethanolamine
Tryptophan
PG(18:1/18:1)
2-Acetylpyrazine
TMP
N-Acetyl-5-hydroxytryptamine
LPC(18:2)
Creatine
3-FORMYLINDOLE
Hordenine
1alpha,25-Dihydroxyvitamin D3
MX1
MX2
MX3
MX4
MX5
MX6
SHR1
SHR2
SHR3
SHR4
SHR5
SHR6
Class
Log2(FC)
-Lg(p)
VIP
Scaled Intensity
Class
AGEs
Amines,choline, and organonitrogen compounds
Amino acids, peptides, and analogues
Fatty acids
Flavonoids, benzene and substituted derivatives
Indoles and heterocyclic compounds
nucleosides,nucleotides, and analogues
Organic acids and derivatives
Phosphatidylacids
Sphingolipids
Vitamins and derivatives
Log2(FC)
-Lg(p)
Group
MX
SHR

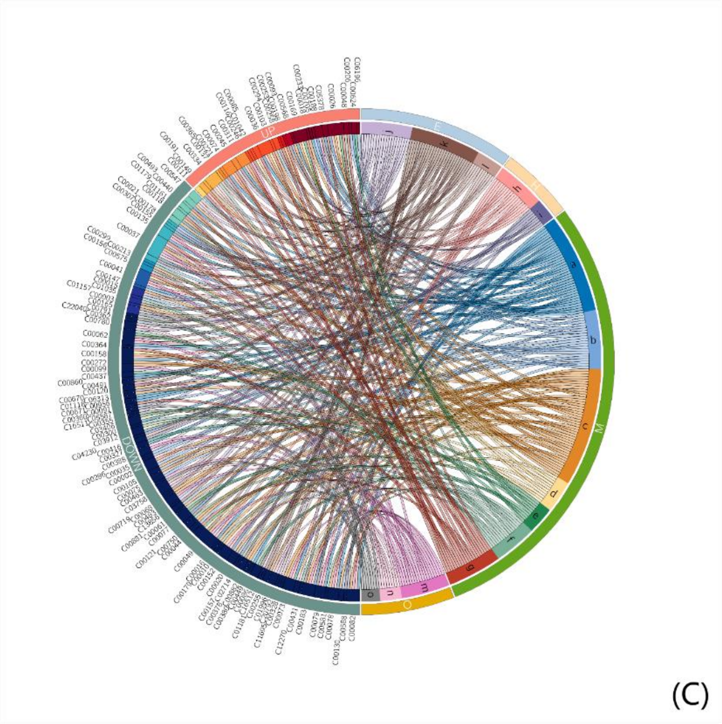

Figure 6. (A) FC and VIP bubble plots of the Model.vs.SHR; (B) Hierarchical clustering results of differential metabolites in the Model.vs.SHR (only the top 50 metabolites with the highest VIP values are plotted); (C) Chord diagram of enriched pathways of the top 15 differential metabolites in the Model.vs.SHR

Table 2. Model vs. SHR group KEGG pathways (Top 15)

Description	pvalue	Count	UP	DOWN
ABC transporters	1.99628E-10	26	6	20
Alanine, aspartate and glutamate metabolism	7.85782E-10	12	5	7
Arginine and proline metabolism	3.51685E-08	16	2	14
Arginine biosynthesis	1.83608E-08	10	3	7
Biosynthesis of amino acids	3.25756E-21	37	10	27
Biosynthesis of cofactors	1.15039E-11	44	10	34
cAMP signaling pathway	4.84679E-08	10	1	9

Carbon metabolism	1.00841E-07	20	13	7
Central carbon metabolism in cancer	2.16204E-15	18	6	12
Choline metabolism in cancer	2.12297E-09	8	1	7
Dopaminergic synapse	2.17561E-07	7	0	7
Neuroactive ligand-receptor interaction	3.30192E-13	19	2	17
Nucleotide metabolism	9.75555E-16	22	5	17
Protein digestion and absorption	3.75657E-13	18	2	16
Synaptic vesicle cycle	6.12459E-09	8	1	7

2.4 Comparison of differential proteins in rat brain tissues in each group

2.4.1 Expression and functional analysis of differential proteins between Model group and WKY group

Compared with the WKY group, there were 141 differentially expressed proteins in the Model group, of which 55 were up-regulated and 86 were down-regulated, see Figure 7 A. The differential proteins between the Model group and the WKY group are enriched in 87 pathways. According to the level 1 level, 30 (34%) are enriched in human diseases, 19 (22%) in biological systems, 18 (21%) in metabolism, and environmental information. There are 12 processes (14%), 4 cellular processes (5%), 3 genetic information processes (3%), and 1 pathway has no attribution, see Figure 7 B. A network of significantly enriched pathways and protein/protein coding genes and protein interactions in the pathway was constructed, including a total of 32 proteins, 12 (+), and 20 expressed (-), as shown in Figure 7 C.

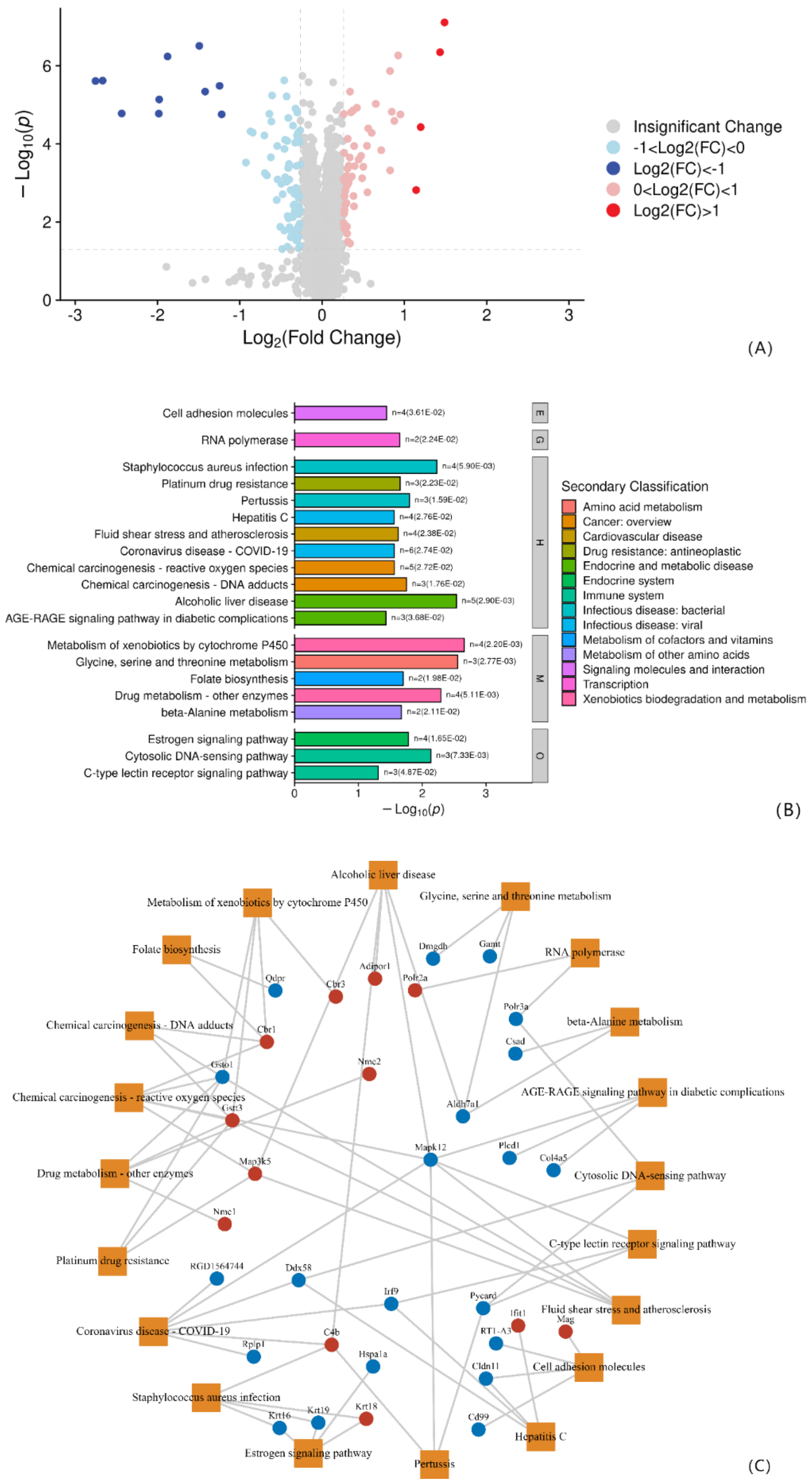

−Log10(p)
Log2(Fold Change)
Insignificant Change
-1<Log2(FC)<0
Log2(FC)<-1
0<Log2(FC)<1
Log2(FC)>1
(A)
Cell adhesion molecules
RNA polymerase
Staphylococcus aureus infection
Platinum drug resistance
Pertussis
Hepatitis C
Fluid shear stress and atherosclerosis
Coronavirus disease - COVID-19
Chemical carcinogenesis - reactive oxygen species
Chemical carcinogenesis - DNA adducts
Alcoholic liver disease
AGE-RAGE signaling pathway in diabetic complications
Metabolism of xenobiotics by cytochrome P450
Glycine, serine and threonine metabolism
Folate biosynthesis
Drug metabolism - other enzymes
beta-Alanine metabolism
Estrogen signaling pathway
Cytosolic DNA-sensing pathway
C-type lectin receptor signaling pathway
n=4(3.61E-02)
n=2(2.24E-02)
n=4(5.90E-03)
n=3(2.23E-02)
n=3(1.59E-02)
n=4(2.76E-02)
n=4(2.38E-02)
n=6(2.74E-02)
n=5(2.72E-02)
n=3(1.76E-02)
n=5(2.90E-03)
n=3(3.68E-02)
n=4(2.20E-03)
n=3(2.77E-03)
n=2(1.98E-02)
n=4(5.11E-03)
n=2(2.11E-02)
n=4(1.65E-02)
n=3(7.33E-03)
n=3(4.87E-02)
E
G
H
M
O
−Log10(p)
Secondary Classification
Amino acid metabolism
Cancer: overview
Cardiovascular disease
Drug resistance: antineoplastic
Endocrine and metabolic disease
Endocrine system
Immune system
Infectious disease: bacterial
Infectious disease: viral
Metabolism of cofactors and vitamins
Metabolism of other amino acids
Signaling molecules and interaction
Transcription
Xenobiotics biodegradation and metabolism
(B)
Alcoholic liver disease
Metabolism of xenobiotics by cytochrome P450
Glycine, serine and threonine metabolism
Folate biosynthesis
RNA polymerase
Chemical carcinogenesis - DNA adducts
beta-Alanine metabolism
Chemical carcinogenesis - reactive oxygen species
AGE-RAGE signaling pathway in diabetic complications
Drug metabolism - other enzymes
Cytosolic DNA-sensing pathway
C-type lectin receptor signaling pathway
Platinum drug resistance
Fluid shear stress and atherosclerosis
Coronavirus disease - COVID-19
Cell adhesion molecules
Staphylococcus aureus infection
Hepatitis C
Estrogen signaling pathway
Pertussis
Dmgdh
Gamt
Adipor1
Qdpr
Cbr3
Polr2a
Polr3a
Cbr1
Csad
Gsto1
Nme2
Gstt3
Aldh7a1
Mapk12
Pltd1
Col4a5
Map3k5
Nme1
RGD1564744
Ddx58
Irf9
Pycard
Ifit1
Mag
RT1-A3
C4b
Rplp1
Hspa1a
Cldn11
Krt16
Krt19
Krt18
Cd99
(C)

Figure 7. (A) Hierarchical clustering results of differential proteins in the Model.vs.WKY; (B) Differential protein enrichment bar chart in the Model.vs.WKY; (C) Significantly enriched pathways and those in the pathway Protein/protein coding genes and protein interaction network

2.4.2 Expression and functional analysis of differential proteins between SHR group and Model group

Compared with the SHR group, there were 28 differentially expressed proteins in the Model group, of which 15 were up-regulated and 13 were down-regulated, see Figure 8 A. Differential proteins between the SHR group and the Model group are enriched in 24 pathways. According to the level 1 level, 9 pathways (38%) are enriched in human diseases, 6 pathways (25%) in biological systems, 3 pathways (13%) in metabolism, and environmental information. There are 3 processing items (13%), 2 cellular process items (8%), and 1 genetic information processing item (4%), see Figure 8 B. A network of significantly enriched pathways and protein/protein coding genes and protein interactions in the pathway was constructed. There were 5 proteins in total, 4 expressed (+) and 1 expressed (-), as shown in Figure 8 C.

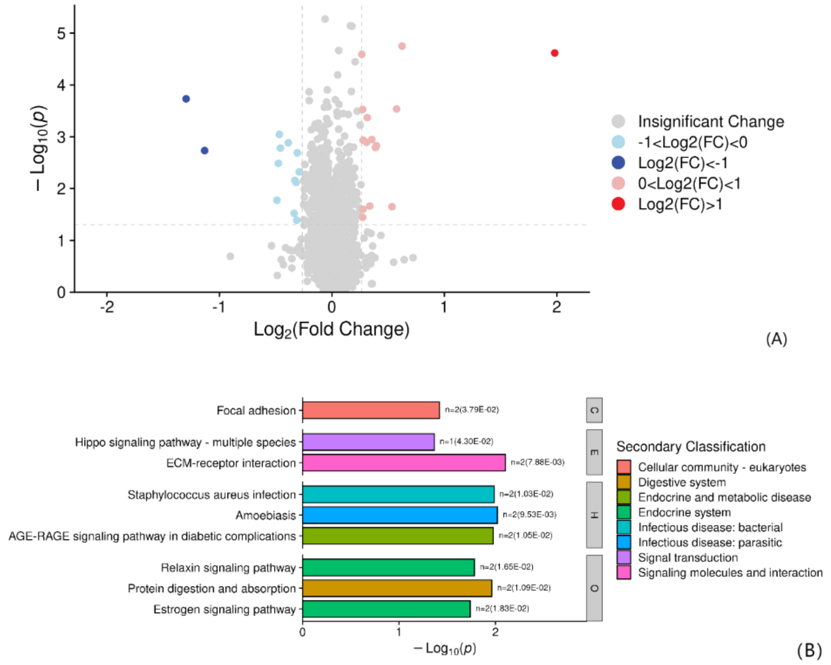

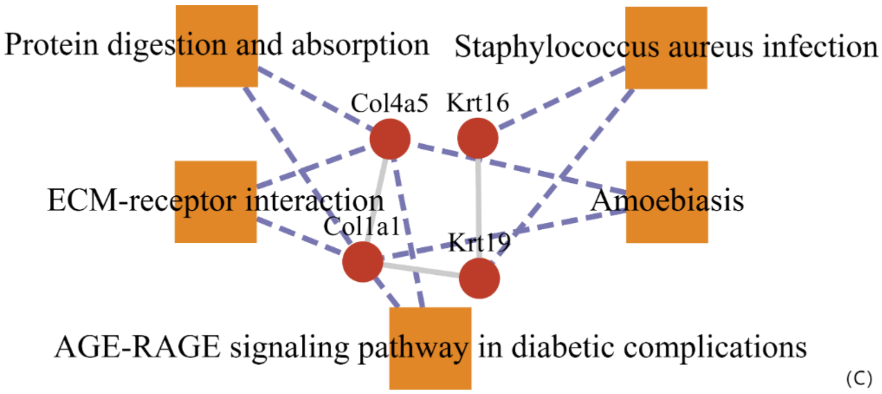

Figure 8. (A) Hierarchical clustering results of differential proteins in SHR vs Model; (B) Differential protein enrichment bar chart in SHR vs Model; (C) Significantly enriched pathways and proteins/proteins in the pathway Coding gene and protein interaction network

3. Discussion

Vascular cognitive impairment is caused by damage to vascular units due to multiple risk factors (hypertension, hyperlipidemia, hyperglycemia, etc.), which ultimately leads to VD[8]. Therefore, the VCI model in which micro-blood flow stimulates blood clots to block the blood vessels and causes cerebral ischemia and hypoxia simulates the clinical pathogenesis and pathophysiological characteristics. In the past, vascular occlusion, intravascular embolism and spontaneous VCI models have been widely used in scientific research. Washida[9] et al. found that asymmetric ligation of bilateral common carotid arteries (the right common carotid artery was implanted with an amilo constrictor to gradually occlude the blood vessels within 28 days, and the left side was implanted with a microcoil to cause about 50% arterial stenosis) can cause chronic cerebral hypoperfusion, cerebral white matter infarction and gliosis in rats. Xue et al.[10] proved that after injection of microsphere vascular embolic agents into the internal carotid artery of SD rats, cerebral blood flow was reduced, escape latency was shortened and the number of perforations was reduced. Spontaneously hypertensive rats are an animal model of chronic

hypertension and persistent susceptibility to ischemic/hypoxic injury. Previous studies have found that the gray matter volume and immunoreactive area of the CA1 subregion and dentate gyrus of SHR rats are reduced, the expression of the cytoskeletal component neurofilament protein is reduced, and astrocytes proliferate[11]. The above findings indicate that the brain tissue of SHR rats has undergone degenerative changes and developed an astrocyte reaction similar to that of VCI. However, simply reducing cerebral perfusion or simply imitating risk factors and waiting for it to spontaneously occur cannot fully simulate the complex pathological process of VCI. Therefore, the research team used the SHR+microcurrent stimulation of bilateral common carotid arteries to induce endothelial injury and thrombosis modeling method, combined with the risk factors and pathogenesis of VCI to replicate a comprehensive animal model.

The main manifestation of VCI is the slow and gradual decline of mental functions including memory, judgment and learning ability. Behavioral comparisons between rats in each group found that from D1 to D5, compared with WKY, the latency of the Model group and SHR group was shorter, the average swimming speed increased, and there was statistical significance ($P<0.01$). This may be related to the type of rat itself. WKY rats showed behavioral defects in multiple behavioral tests, especially in long-term darkness. Under the environment, their autonomous activities were significantly weakened, and in comparison, SHR rats had stronger activity abilities. On D6, the platform was removed and the spatial exploration test was started. Compared with the WKY group, the number of platform crossings in the Model group was significantly reduced, and the search time for the target quadrant and the total distance to the target quadrant were reduced ($P<0.05$), which suggested the spatial learning and memory ability of the rats in the model group. Injury, SHR + microcurrent stimulation of bilateral common carotid arteries can simulate the clinical characteristics of cognitive dysfunction in patients with VCI. However, there is no statistical significance in the behavioral comparison between the SHR

group and the Model group. SHR rats are similar to human essential hypertension, with normal blood pressure at birth and gradually developing into stable blood pressure within the first 2-4 months after birth. of hypertension [12]. In 4-month-old SHR rats, a limited reduction in white matter volume can be observed. In 6-month-old SHR rats, the volume of hippocampal CA1 region and dentate gyrus gray matter is reduced, the expression of glial fibrillary acid protein is increased, and the number of astrocytes is increased. In this study, 13-14-week-old SHR rats were selected, and the samples were collected 1 week after modeling, which shows that SHR rats can already show similar manifestations of vascular cognitive impairment. Therefore, compared with the WKY group, the number of platform crossings, target quadrant search time and distance in the SHR group were reduced ($P>0.05$), and there was no statistical significance between the SHR group and the Model group. HE staining was used to observe the pathological state of brain tissue in rats in each group, and Nissl staining was used to observe the state of neuronal Nissl bodies. The results of HE staining showed that compared with the WKY group, the number of neurons in the hippocampal tissue structure of rats in the SHR group and the Model group was reduced, the nuclei were deformed and condensed, the staining was deepened, and the cell bodies were relatively disordered. The results of Nissl staining showed that the hippocampal neurons in the SHR group and the Model group were irregularly arranged and sparsely distributed, with lighter Nissl staining, and the morphology of some hippocampal neurons in the Model group changed from granular to powdery, with pyknosis of the cell nucleus, accompanied by cell edema and necrosis, which indicated that the model was successfully established.

VCI and VD have been found to be associated with metabolic changes [13]. Further elucidation of the pathophysiological characteristics of VCI will help reduce its high incidence. In this study, we used a method combining TMT quantitative proteomics technology with liquid

chromatography tandem mass spectrometry (LC-MS/MS) to analyze metabolomics and proteomics data in brain tissue samples to reveal changes in metabolites and proteins in VCI. Based on a multi-omics joint analysis method, a reliable network for the systematic biological analysis of VCI from proteins to final metabolites was constructed.

Amino acid metabolism disorder is one of the factors that lead to the formation of VD[13]. Phenylalanine and tryptophan are essential amino acids for the human body. Studies have found that phenylalanine can induce oxidative stress and reduce the activity of rat astrocytes. Compared with the sham operation group, the expression of phenylalanine in Aβ1-42-induced rat hair samples was upregulated[14]. However, some studies have also shown that lower concentrations of phenylalanine were detected in the serum of patients with cognitive dysfunction[15]. The expression of phenylalanine in brain tissue of the model group in this study was downregulated, so there is still controversy about the expression of phenylalanine in VD, which may also be related to the different test samples selected in each study. Tryptophan intervenes in the function of the central nervous system by regulating indole derivatives, kynurenine pathways and serotonin synthesis. Its concentration is related to the onset of chronic cerebral ischemia, neuronal damage and neurodegenerative diseases[16,17]. Citrulline acetate is a precursor of creatine. Creatine can increase the energy supply of brain cells, especially in the form of creatine phosphate, maintain the ATP level of brain cells, and enhance memory by increasing the synthesis of neurotransmitters such as acetylcholine and reducing oxidative stress damage to brain cells [18]. Metabolomics results showed that compared with the SHR group and the WKY group, the expression of tryptophan, citrulline acetate, and creatine in the Model group was downregulated, which is similar to the results of previous studies.

Excessive activation of excitatory amino acids such as glutamate is considered an important link in neuronal damage [19,20]. Compared with the control group, the differential metabolites in

the Model group were mainly concentrated in amino acid metabolism pathways such as alanine, aspartic acid and glutamic acid metabolism, arginine and proline metabolism, and arginine biosynthesis, among which Aspartate and glutamate are both excitatory amino acids. N-acetyl aspartate glutamate (NAAG) is a ubiquitous neuropeptide in the central nervous system and is related to presynaptic II metabotropic glutamate. Receptor subtype 3 binds and inhibits glutamate release [21]. In this study, the decreased expression of NAAG will increase the release of glutamate, accumulate in the synaptic cleft or even overflow, leading to excessive activation of extrasynaptic N-methyl-D-aspartate receptors (NMDAR) and causing synaptic plasticity. impairment and excitotoxicity [22]. Mitochondrial dysfunction, oxidative stress, etc. promote the development of cognitive dysfunction, and arginine significantly attenuates the generation of mitochondrial reactive oxygen species induced by angiotensin II [23]. Arginine is decomposed into nitric oxide under the action of nitric oxide synthase. Nitric oxide can serve as a neurotransmitter, mediating excitatory amino acids and synaptic transmission. The production and excessive release of nitric oxide can directly lead to neurotoxicity [24]. The proteomics differential proteins were also enriched in amino acid metabolism, confirming the metabolomics results.

In addition, compared with the SHR group, the levels of thiamine monophosphate (TMP) and vitamin B1 in the Model group decreased, which is similar to previous studies [25]. TMP is an intermediate form in the process of vitamin B1 being converted to thiamine pyrophosphate (TPP) in the body. TPP is a coenzyme of key enzyme systems such as the α-ketoacid oxidative decarboxylase multienzyme complex and is essential for glucose metabolism. The reduction of TMP and TDP may lead to reduced glucose metabolism in brain tissue, the formation of neuritic plaques and hyperphosphorylation of tau protein, and promote the occurrence of cognitive dysfunction [25,26].

Relaxin can affect brain memory by inhibiting the synthesis of cAMP and reducing the activity level of the cAMP-PKA pathway [27]. It can also reduce brain inflammation to improve white matter loss, reduced cortical thickness and cognitive dysfunction [28]. Focal adhesions are physical contact points between the extracellular matrix and the cell actin cytoskeleton [29]. In the nervous system, the connection and adhesion between neurons are crucial for the transmission of information and the maintenance of cognitive function [30]. Impaired function of focal adhesions may affect the normal connection and communication of neurons, thereby having a negative impact on cognitive function. Previous metabolomics studies have also suggested that focal adhesions play a role in cognitive dysfunction [31]. In this study, the differentially expressed proteins between the SHR group and the Model group were enriched in the "Relaxin Signaling Pathway" and "Focal Adhesion" pathways, which also confirms that relaxin and focal adhesions have a certain influence on the development of hypertension to vascular dementia.

In summary, the VCI rat model caused by hypertension combined with endothelial injury established in this study simulates the clinical characteristics of cognitive dysfunction caused by risk factors of VCI and is an ideal animal model. Compared with the spontaneous SHR model, the SHR+microcurrent stimulation of bilateral common carotid arteries to cause endothelial injury and thrombosis can simulate vascular injury under hypertension, thereby causing changes in cerebral hemodynamics and hypoxia, ischemia and other damage to brain tissue, which are important pathological mechanisms of VCI and VD. Therefore, this animal model is closer to the pathological process of human VCI and VD and enables the model to show VCI and even characteristic pathological changes of VD more quickly. In addition, the application of metabolomics and proteomics has important guiding value in studying the diagnostic indicators and therapeutic targets of VCI and VD patients. VCI and VD mediated by hypertension as a risk

factor mainly involve pathways such as amino acid metabolism, relaxin signaling pathway and focal adhesion. Metabolites such as TMP and vitamin B1 also play a certain role in the occurrence and development of VD. Although there are differences between animal models and VCI patients, this analysis also shows a high degree of consistency with the information of VCI and VD patients [32]. This study analyzed valuable biomarkers and related metabolic pathways, which can also provide a reference for the pathogenesis of VCI and VD. This study still has the following defects: Although the uniformity of SPF-level experimental animals is good, more behavioral tests are needed as support. The research of the research team is still preliminary. In future studies, molecular biological methods should be used to verify the signal transduction pathways, and combined with human samples (such as serum) of patients for verification, so as to screen out accurate and efficient biomarkers.

Conclusion

Rats with hypertension combined with endothelial injury have behavioral disorders, which are similar to the causes and signs of clinical vascular cognitive impairment; the biological mechanisms manifested in the model involve amino acid metabolism disorders, relaxin signaling pathway and impaired focal adhesion function.

Acknowledgments

The combined analysis of metabolomics and proteomics in this project was assisted by Engineer Du Dan of Shanghai Baipu Biotechnology Co., Ltd., and we would like to express our sincere gratitude!

Authors’ contributions

Xiao Zhang，Surui Chang，Jiangang Liu designed and conceived the study; Xiao Zhang, Surui Chang and Xiaoyuan Wang performed the research; Xiao Zhang and Jiangang Liu participated in the results discussion and technical support; Xiao Zhang and Surui Chang wrote

the original draft; Jiangang Liu review and edit the paper. All authors read and approved the fnal manuscript.

Funding

This study was supported by Major Increase and Decrease Projects at Central Level (No.2060302).

Ethics approval and consent to participate

During the experiment, the treatment of animals complied with the ethical requirements ou tlined in the "Regulations on the Administration of Experimental Animals" by the National Scie nce and Technology Commission and the "Guidelines for the Welfare and Ethical Review of Ex perimental Animals in Beijing." The study was approved by the Ethics Committee of Xiyuan H ospital, China Academy of Chinese Medical Sciences (№: 2021XLC002-2). This research was done in compliance with the ARRIVE guidelines and regulations (https://arriveguidelines.org).

Consent for publication

Not applicable.

Competing interests

The authors declare that they have no competing interests

Data availability

Data is provided within the supplementary information files.

Reference

[1] Chinese Stroke Society Vascular Cognitive Impairment Branch. Chinese guidelines for the diagnosis and treatment of vascular cognitive impairment (2024 edition)[J/OL]. Chinese Medical Journal, 2024, 104(0): 1-14. DOI:10.3760/cma.j.cn112137-20240501-01024.

[2] CAO Q, TAN C C, XU W, etc.. The Prevalence of Dementia: A Systematic Review and Meta-Analysis[J/OL]. Journal of Alzheimer's disease: JAD, 2020, 73(3): 1157-1166. DOI:10.3233/JAD-191092.

[3] LINH T T D, HSIEH Y C, HUANG L K, etc.. Clinical Trials of New Drugs for Vascular Cognitive Impairment and Vascular Dementia[J/OL]. International Journal of Molecular Sciences, 2022, 23(19): 11067. DOI:10.3390/ijms231911067.

[4] MORGAN A E, MC AULEY M T. Vascular dementia: From pathobiology to emerging perspectives[J/OL]. Ageing Research Reviews, 2024, 96: 102278. DOI:10.1016/j.arr.2024.102278.

[5] Qun Wang, Yongjun Wang. Rodent models of vascular dementia[J]. Chinese Journal of Stroke, 2015, 10(4): 279-283.

[6] LEVINE D A, SPRINGER M V, BRODTMANN A. Blood Pressure and Vascular Cognitive Impairment[J/OL]. Stroke, 2022, 53(4): 1104-1113. DOI:10.1161/STROKEAHA.121.036140.

[7] NAGAI M, HOSHIDE S, KARIO K. Hypertension and Dementia[J/OL]. American Journal of Hypertension, 2010, 23(2): 116-124. DOI:10.1038/ajh.2009.212.

[8] RUNDEK T, TOLEA M, ARIKO T, etc.. Vascular Cognitive Impairment (VCI)[J/OL]. Neurotherapeutics, 2022, 19(1): 68-88. DOI:10.1007/s13311-021-01170-y.

[9] WASHIDA K, HATTORI Y, IHARA M. Animal Models of Chronic Cerebral Hypoperfusion: From Mouse to Primate[J/OL]. International Journal of Molecular Sciences, 2019, 20(24): 6176. DOI:10.3390/ijms20246176.

[10] XUE B, MA B, YAO Y, etc.. BYHW Decoction Improves Cognitive Impairments in Rats with Cerebral Microinfarcts via Activation of the PKA/CREB Pathway[J/OL]. Oxidative Medicine and Cellular Longevity, 2022, 2022: 1-21. DOI:10.1155/2022/4455654.

[11] SABBATINI M, CATALANI A, CONSOLI C, etc.. The hippocampus in spontaneously

hypertensive rats: an animal model of vascular dementia?[J/OL]. Mechanisms of Ageing and Development, 2002, 123(5): 547-559. DOI:10.1016/S0047-6374(01)00362-1.

[12]STEPANICHEV M Y, MAMEDOVA D I, GULYAEVA N V. Hippocampus under Pressure: Molecular Mechanisms of Development of Cognitive Impairments in SHR Rats[J/OL]. Biochemistry. Biokhimiia, 2024, 89(4): 711-725. DOI:10.1134/S0006297924040102.

[13]CHUMACHENKO M S, WASEEM T V, FEDOROVICH S V. Metabolomics and metabolites in ischemic stroke[J/OL]. Reviews in the Neurosciences, 2022, 33(2): 181-205. DOI:10.1515/revneuro-2021-0048.

[14]TAN T H, LI S W, CHANG C W, etc.. Rat Hair Metabolomics Analysis Reveals Perturbations of Unsaturated Fatty Acid Biosynthesis, Phenylalanine, and Arachidonic Acid Metabolism Pathways Are Associated with Amyloid-β-Induced Cognitive Deficits[J/OL]. Molecular Neurobiology, 2023, 60(8): 4373-4395. DOI:10.1007/s12035-023-03343-6.

[15]VAN DER VELPEN V, TEAV T, GALLART-AYALA H, etc.. Systemic and central nervous system metabolic alterations in Alzheimer’s disease[J/OL]. Alzheimer’s Research & Therapy, 2019, 11(1): 93. DOI:10.1186/s13195-019-0551-7.

[16]LIAO W, XUE Z, WANG X, etc.. Metabolic profiling deciphering the potential targets of Yi-Gan San against vascular dementia in rat[J/OL]. Brain Research, 2020, 1727: 146512. DOI:10.1016/j.brainres.2019.146512.

[17]WU L, HAN Y, ZHENG Z, etc.. Altered Gut Microbial Metabolites in Amnestic Mild Cognitive Impairment and Alzheimer’s Disease: Signals in Host–Microbe Interplay[J/OL]. Nutrients, 2021, 13(1): 228. DOI:10.3390/nu13010228.

[18]XU C, BI S, ZHANG W, etc.. The effects of creatine supplementation on cognitive function in adults: a systematic review and meta-analysis[J/OL]. Frontiers in Nutrition, 2024, 11: 1424972. DOI:10.3389/fnut.2024.1424972.

[19]BARANOVICOVA E, KALENSKA D, KAPLAN P, etc.. Blood and Brain Metabolites after Cerebral Ischemia[J/OL]. International Journal of Molecular Sciences, 2023, 24(24): 17302. DOI:10.3390/ijms242417302.

[20]KUMAR M, KUMAR A, SINDHU R K, etc.. Arbutin attenuates monosodium L-glutamate induced neurotoxicity and cognitive dysfunction in rats[J/OL]. Neurochemistry International, 2021, 151: 105217. DOI:10.1016/j.neuint.2021.105217.

[21]GURKOFF G G, FENG J F, VAN K C, etc.. NAAG peptidase inhibitor improves motor function and reduces cognitive dysfunction in a model of TBI with secondary hypoxia[J/OL]. Brain Research, 2013, 1515: 98-107. DOI:10.1016/j.brainres.2013.03.043.

[22]DU K, ZHAI C, LI X, etc.. Feature-Based Molecular Networking Facilitates the Comprehensive Identification of Differential Metabolites in Diabetic Cognitive Dysfunction Rats[J/OL]. Metabolites, 2023, 13(4): 538. DOI:10.3390/metabo13040538.

[23]MONE P, PANSINI A, JANKAUSKAS S S, etc.. L-Arginine Improves Cognitive Impairment in Hypertensive Frail Older Adults[J/OL]. Frontiers in Cardiovascular Medicine, 2022, 9: 868521. DOI:10.3389/fcvm.2022.868521.

[24]KIM S H, JU I G, KIM J H, etc.. Linderae Radix Ameliorates Cognitive Dysfunction by Inhibiting Neuroinflammation and Synaptic Damage in Alzheimer’s Disease Models[J/OL]. Molecular Neurobiology, 2023, 60(12): 7196-7207. DOI:10.1007/s12035-023-03544-z.

[25]PAN X, FEI G, LU J, etc.. Measurement of Blood Thiamine Metabolites for Alzheimer’s Disease Diagnosis[J/OL]. EBioMedicine, 2016, 3: 155-162. DOI:10.1016/j.ebiom.2015.11.039.

[26]PAN X, SANG S, FEI G, etc.. Enhanced Activities of Blood Thiamine Diphosphatase and Monophosphatase in Alzheimer’s Disease[J/OL]. PLOS ONE, 2017, 12(1): e0167273. DOI:10.1371/journal.pone.0167273.

[27]GIL-MIRAVET I, MAÑAS-OJEDA A, ROS-BERNAL F, etc.. Involvement of the Nucleus

Incertus and Relaxin-3/RXFP3 Signaling System in Explicit and Implicit Memory[J/OL]. Frontiers in Neuroanatomy, 2021, 15: 637922. DOI:10.3389/fnana.2021.637922.

[28]XU M M, SEYLER L, BÄUERLE T, etc.. Serelaxin activates eNOS, suppresses inflammation, attenuates developmental delay and improves cognitive functions of neonatal rats after germinal matrix hemorrhage[J/OL]. Scientific Reports, 2020, 10(1): 8115. DOI:10.1038/s41598-020-65144-4.

[29]FABRY B, KLEMM A H, KIENLE S, etc.. Focal adhesion kinase stabilizes the cytoskeleton[J/OL]. Biophysical Journal, 2011, 101(9): 2131-2138. DOI:10.1016/j.bpj.2011.09.043.

[30]SUO L, LU H, YING G, etc.. Protocadherin clusters and cell adhesion kinase regulate dendrite complexity through Rho GTPase[J/OL]. Journal of Molecular Cell Biology, 2012, 4(6): 362-376. DOI:10.1093/jmcb/mjs034.

[31]Y C, T L, Z L, etc.. Neurofilament degradation is involved in laparotomy-induced cognitive dysfunction in aged rats[J/OL]. Aging, 2020, 12(24)[2024-08-01]. https://pubmed.ncbi.nlm.nih.gov/33232265/. DOI:10.18632/aging.104172.

[32]HARSHFIELD E L, SANDS C J, TULADHAR A M, etc.. Metabolomic profiling in small vessel disease identifies multiple associations with disease severity[J/OL]. Brain: A Journal of Neurology, 2022, 145(7): 2461-2471. DOI:10.1093/brain/awac041.

Click here to access/download

Supporting Information - Compressed/ZIP File Archive

figures.zip
