# Supplementary material for: Combined metabolomics and proteomics analysis of vascular cognitive impairment in hypertensive rats induced by endothelial injury
Source: PLoS One. 2025 Oct 9;20(10):e0332827. doi: 10.1371/journal.pone.0332827 (PMC12510566; doi:10.1371/journal.pone.0332827)
Supplement: Attachment — Submitted filename: Response to Reviewers.doc [file pone.0332827.s008.doc]

We would like to express our sincere gratitude to the editor and the reviewers for their conscientiousness and responsibility. They promptly pointed out the issues in our article and kindly provided us with an opportunity for revision. We have polished the language of the article and made modifications to the images and layout. We sincerely hope that these revisions will meet your satisfaction.

**Journal Requirements:**

1. Please ensure that your manuscript meets PLOS ONE's style requirements, including those for file naming. The PLOS ONE style templates can be found at

https://journals.plos.org/plosone/s/file?id=wjVg/PLOSOne_formatting_sample_main_body.pdf and

https://journals.plos.org/plosone/s/file?id=ba62/PLOSOne_formatting_sample_title_authors_affiliations.pdf

Response: The manuscript has been revised in accordance with the formatting requirements of PLOS ONE.

2. To comply with PLOS ONE submissions requirements, in your Methods section, please provide additional information regarding the experiments involving animals and ensure you have included details on (1) methods of sacrifice, and (2) efforts to alleviate suffering.

Response: During the rat modeling process, sodium pentobarbital was administered via intraperitoneal injection for anesthesia to alleviate the suffering of the rats. Euthanasia of the rats was performed by intravenous injection of an excessive dose of sodium pentobarbital. The relevant descriptions have been added to the article.

3. Thank you for stating the following financial disclosure: This study was supported by Major Increase and Decrease Projects at Central Level (No.2060302). Please state what role the funders took in the study. If the funders had no role, please state: ""The funders had no role in study design, data collection and analysis, decision to publish, or preparation of the manuscript.""

If this statement is not correct you must amend it as needed.

Please include this amended Role of Funder statement in your cover letter; we will change the online submission form on your behalf.

Response: Relevant descriptions have been added both in the article and the cover letter.

4. Your ethics statement should only appear in the Methods section of your manuscript. If your ethics statement is written in any section besides the Methods, please move it to the Methods section and delete it from any other section. Please ensure that your ethics statement is included in your manuscript, as the ethics statement entered into the online submission form will not be published alongside your manuscript.

Response: The ethical statement outside the Methods section of the manuscript has been deleted.

5. Please include captions for your Supporting Information files at the end of your manuscript, and update any in-text citations to match accordingly. Please see our Supporting Information guidelines for more information: http://journals.plos.org/plosone/s/supporting-information.

Response: The Supporting Information has been supplemented.

**Reviewer #1:**

1.The language in this submission requires improvement, as some sentences are unclear or awkwardly phrased. Additionally, the sentence arrangement could be better organised for coherence and readability. In the statistical analysis section, the explanation of the validity for OPLS-DA was missing, as were the values of the selected VIP and the criteria used for comparisons. Information about the software used for statistical analysis was also not provided.While comparisons between groups (intergroup comparisons) were mentioned, it is unclear whether intragroup comparisons were performed. This should be clarified. No references were provided for some of the major methods.

Response: The language of the manuscript has been improved. In the statistical section, an explanation of OPLS-DA and the comparison criteria for VIP values have been supplemented. This article conducts comparisons between groups and does not perform comparisons within groups. Relevant references have been added to the methodology section.

2.The total number of rats was stated as 30, with 15 SHR and 15 WKY. However, in Section 1.3, it is mentioned that there are three groups: WKY (10 rats), model (10 rats), and SHR (10 rats). Does the model group comprise 5 SHR and 5 WKY?

Response: The total number of rats is 30, which should be 10 in the WKY group, 10 in the Model group (that is, SHR + microcurrent stimulation group), and 10 in the SHR group. There was an error in the previous description, and thank you for pointing it out.

3.Results and discussion: More discussion is needed and references. It is suggested to provide a diagram to highlight which metabolites are shared and different among those three groups.

Response: The discussion section has been refined, and table related to the main differential metabolites among the three groups have been added.

4.Conclusion: Didn't highlight the major findings in the study—too simple and general.

Response: The conclusion section has been improved.

5.Others: Kindly provide the limitations of the study. Formatting was not consistent throughout the manuscript (example: 1.5.2).

Response: The research limitations have been added to the discussion section, and the article format has been corrected and revised.

**Reviewer #2:**

1.Section 1.8. P<0.05 threshold should be adjusted because of the multiple testing problem to reduce false discovery rate.

Response: For multiple testing, the Bonferroni and Dunnett T3 tests were selected. The P-values obtained from both methods are corrected values.

2.Figure 2. What does the error bar mean? If the error bars mean confidence interval, since many of the overlap with each other, the difference between different groups should not be significant. Hence, the significant levels (***, **) are questionable.

Response: The error bars specifically represent the mean±standard deviation. Other types of charts have been used to make the presentation clearer.

3.What softwares were used to annotate the metabolites? The authors should report the metabolite annotation level of the analysis.

Response: MT1000 Metabolite Identification Software: MultiQuant. It utilizes ion pairs for metabolite identification, and the identification levels are Level 1 and Level 2. It has been indicated in the text.

4.What softwares were used to annotate protein?

Response: Protein Database Search Software for TMT Project: Proteome Discoverer.

5.Figure 5A and 6A. what is the total number of metabolites shown? The abundance table of them should be provided as supplementary material. It seems there are more than 437 and 449 significant metabolites.

Response: The table of abundance of differential metabolites has been supplemented and uploaded.

6.Figure 5C and 6C. They look very confusing. Please describe more.

Response: The chart types of Figure 5C and Figure 6C have been changed to make the pathways clearer.

7.There are a lot of typo and grammar issues. e.g.

Response: The grammar and spelling of the article have been corrected and improved.
