# Supplementary material for: Combined metabolomics and proteomics analysis of vascular cognitive impairment in hypertensive rats induced by endothelial injury
Source: PLoS One. 2025 Oct 9;20(10):e0332827. doi: 10.1371/journal.pone.0332827 (PMC12510566; doi:10.1371/journal.pone.0332827)
Supplement: Attachment — Submitted filename: Response_to_Reviewers_auresp_2.doc [file pone.0332827.s009.doc]

**Editor**

- **Please remove your figures/ from within your manuscript file, leaving only the individual TIFF/EPS image files. These will be automatically included in the reviewer’s PDF.**
- The image has been removed from the manuscript.

**Reviewer #3:**

- Therefore, while it is acceptable that hippocampal pathology was examined in this study, it would be helpful for the authors to clarify that:
  Only the hippocampus was assessed histologically,
  The behavioral evaluation mainly reflects cognitive performance and does not encompass depressive or affective domains,
  The findings should be interpreted within these limitations.
  Moreover, while the metabolomic and proteomic analyses are well conducted, many of the identified molecular changes—particularly those involving neurotransmitters and amino acid metabolism—are broadly linked to aging or psychiatric disorders. Some brief discussion on this point would strengthen the clinical relevance of the findings.
  In summary, I recommend only minor textual revisions, particularly to the limitations and discussion sections, with the goal of making the implications more clinically accessible for practicing physicians. Overall, the manuscript is of high quality and should be considered favorably for publication after these small adjustments.
- Sincerely thank you for your highly constructive and professional comments on this study. You have accurately identified the key areas that need improvement in the study’s evaluation scope and result interpretation, and these comments hold important guiding significance for enhancing the rigor, clinical relevance, and readability of the study. We have carefully organized your suggestions, supplemented the discussion on the study’s limitations, and streamlined the discussion section.

**Reviewer #4:**

- You use the term “model” group, but this is ambiguous, I think actually mean control or sham or equivalent Please amend in all text.
- Thank you very much for your comments. The focus of this study is on proposing a new method for establishing a rat model of vascular cognitive impairment (VCI), and it is considered that "SHR + microcurrent stimulation" is a promising option for VCI model establishment. Therefore, **SHR rats + microcurrent stimulation** were defined as the **Model group**, for comparison with the other two groups. The **WKY group** consists of WKY rats that underwent only common carotid artery dissection without **microcurrent stimulation**; The **SHR group** consists of SHR rats **without any intervention**.
- Figure 1 – surgical images can be useful but the two right hand images are not necessary (microcurrent device and the low magnification rat and frame) – so please remove them. The two right hand images are useful but please add label with arrows and refer to each image in the figure legend. Note, in your methods you write that “neck skin depilated and disinfected with iodine tincture”, but this does not appear to have happen in the images? Please clarify.
- Thank you for your comment. The surgical images have been removed.
- Results 2.1 – Please explain the purpose of the experiment before you describe the data at the beginning of the paragraph, so the reader has context for what is to follow. Two sentences are sufficient.Results 2.2 – Again, please explain the context of the experiment at the beginning of the paragraph before describing the data. This is otherwise quite hard to follow. Two sentences are sufficient.Results 2.4.1 – again please explain the context of the experiment as in 2.1 and 2.2 above.
- Discussions on experimental objectives have been added to Sections 2.1, 2.2 and 2.4.
- Figure 3 – please add the group labels to the images directly for clarity and readability；Figure 4 – please add group labels to the images for clarity.
- Thank you very much for your comment. Group labels have been directly added to the pathological images.
- P22 - Some phrases are confusing in naming. E.g “In this study, SHR rats aged 13 to 14 weeks were selected, and the samples were collected 1 week after modelling” – is this the microcurrent damage? It is confusing that you have the other group called “model”. And on page 27 you refer to another group of “SHR model”. Please clarify in the text in each instance.
- Thank you for your valuable comments. Here, the SHR rats specifically refer to those in the SHR group. We apologize for any confusion caused. Additionally, all mentions of the "SHR group" and "Model group" in the manuscript have been revised to "SHR group (no vascular injury)" and "Model group (SHR+vascular injury composite model)" respectively.
- Discussion Figure 3 – this figure does not seem useful and is also not quantified. If the data is already in the results section, then it should not be duplicated. The name as Figure 3 also does not make sense as there are 7 figures above. Please either remove if it is essential then move it to the results.
- Thank you for your suggestion. The table has been moved to the Results section.
- Discussion text. This is overly speculative with long description of pathways with only passing relevance to the data in the manuscript e.g p26 GABA. Please cut down the discussion to include only those hypotheses most strongly supported by the data in this manuscript and the wider literature.
- Sincerely thank you for your incisive comments and constructive suggestions on the Discussion section of this study. Regarding this issue, the Discussion section has undergone systematic streamlining and restructuring: specifically, we have eliminated subjective speculations, condensed redundant pathway descriptions, and retained only the hypotheses supported by the data of this study.
- In a previous comment the authors say “All relevant data are within the manuscript and its Supporting Information files” however this does not appear to be in the submission. Please provide all raw data for this manuscript
- Thank you for your reminder. The data have been supplemented.

**Reviewer #5:**

- The authors responded to the the comments with care and diligence, making substantial revisions that improved the clarity, rigor, and overall quality of the manuscript. Methodological details were clarified, additional analyses were added, and key sections were rewritten for precision and coherence. The manuscript is now suitable for publication and makes a valuable contribution to the field.
- Sincerely thank you for your recognition and affirmation of the revision work of this study! It is precisely based on your previous professional comments that we have been able to targetedly clarify the methodological details, ultimately enabling the paper to better align with academic standards and better highlight its research value. Once again, we extend our most sincere gratitude to you! Your professional guidance has been key support for improving the quality of this study, and has also set a benchmark for rigorous academic research in our future scientific work.
